# Modeling autosomal dominant optic atrophy using induced pluripotent stem cells and identifying potential therapeutic targets

**DOI:** 10.1186/s13287-015-0264-1

**Published:** 2016-01-07

**Authors:** Jing Chen, Hamidreza Riazifar, Min-Xin Guan, Taosheng Huang

**Affiliations:** Division of Human Genetics, Cincinnati Children’s Hospital Medical Center, Cincinnati, OH 45229 USA; Department of Pediatrics, Division of Human Genetics, University of California, Irvine, CA 92697 USA

**Keywords:** Optic atrophy, OPA1, Induced pluripotent stem cells, DAPT, Noggin, β-estrogen

## Abstract

**Background:**

Many retinal degenerative diseases are caused by the loss of retinal ganglion cells (RGCs). Autosomal dominant optic atrophy is the most common hereditary optic atrophy disease and is characterized by central vision loss and degeneration of RGCs. Currently, there is no effective treatment for this group of diseases. However, stem cell therapy holds great potential for replacing lost RGCs of patients. Compared with embryonic stem cells, induced pluripotent stem cells (iPSCs) can be derived from adult somatic cells, and they are associated with fewer ethical concerns and are less prone to immune rejection. In addition, patient-derived iPSCs may provide us with a cellular model for studying the pathogenesis and potential therapeutic agents for optic atrophy.

**Methods:**

In this study, iPSCs were obtained from patients carrying an *OPA1* mutation (*OPA1*^*+/−*^*-*iPSC) that were diagnosed with optic atrophy. These iPSCs were differentiated into putative RGCs, which were subsequently characterized by using RGC-specific expression markers BRN3a and ISLET-1.

**Results:**

Mutant *OPA1*^*+/−*^-iPSCs exhibited significantly more apoptosis and were unable to efficiently differentiate into RGCs. However, with the addition of neural induction medium, Noggin, or estrogen, *OPA1*^*+/−*^-iPSC differentiation into RGCs was promoted.

**Conclusions:**

Our results suggest that apoptosis mediated by *OPA1* mutations plays an important role in the pathogenesis of optic atrophy, and both noggin and β-estrogen may represent potential therapeutic agents for *OPA1-*related optic atrophy.

**Electronic supplementary material:**

The online version of this article (doi:10.1186/s13287-015-0264-1) contains supplementary material, which is available to authorized users.

## Background

Autosomal dominant optic atrophy (DOA) is the most common hereditary optic atrophy and is characterized by central vision loss and degeneration of retinal ganglion cells (RGCs). RGCs transmit visual information from photoreceptors in the eye to several regions in the thalamus, hypothalamus, and midbrain. The axons from RGCs also form the optic nerve and extend to the optic chiasm and visual cortex. The optic nerve is vulnerable to genetic and environmental risks, and *OPA1* mutations are the most common cause of DOA. *OPA1* is a nuclear gene that encodes an inner mitochondrial membrane protein. Mutations in *OPA1*, including missense, nonsense, deletions/insertions, and splicing mutations, have resulted in decreased ATP production, reduced mitochondrial membrane potential, and mitochondrial dysfunction, including mitochondrial fragmentation [1–3]. Some *OPA1* mutations have also resulted in truncated mutant proteins and therefore haploinsufficiency is responsible for certain clinical phenotypes. Unfortunately, a majority of optic atrophy patients have experienced significant RGC loss by the time of diagnosis. Therefore, cellular therapy may be a promising treatment, especially given the advances that have been made in the use of human embryonic stem cells (hESCs) and induced pluripotent stem cells (iPSCs) [4–9].

iPSCs are derived from nonpluripotent adult cells that have various genes and transcription factors induced [10, 11]. As a result, iPSCs and hESCs share many defining characteristics: they both express various stem cell-specific markers, they maintain a self-renewal capacity, and they can be differentiated into various cell types. Correspondingly, when iPSCs are injected into mouse blastocysts, viable chimeras have been generated. Although additional research is needed, iPSCs have the potential to be a useful tool for drug screening and disease modeling. In addition, the use of iPSCs for cell therapy may facilitate transplantation approaches that are associated with fewer ethical concerns and are less prone to immune rejection compared with that of hESCs.

The differentiation of iPSCs into RGCs is regulated by many intrinsic and extrinsic factors, including those involved in Notch signaling pathways [12, 13]. For example, Notch signaling regulates the total number of RGCs by lateral inhibition of the retina and prevents retinal progenitor cells from differentiating. As a result, retinal progenitor cell status is maintained and RGC production is inhibited [14, 15]. Notch signaling is also downregulated immediately before the differentiation of retinal progenitor cells into RGCs. Therefore, inhibition of Notch signaling may promote RGC production.

Recently, we developed a stepwise chemical protocol for the differentiation of hESCs and normal iPSCs into functional RGCs by using N-[N-(3, 5-difluorophenacetyl)-l-alanyl]-S-phenylglycine t-butyl ester (DAPT) [16]. In the present study, we were able to generate human iPSCs from fibroblast cells obtained from patients diagnosed with optic atrophy and carrying an *OPA1* mutation. The goal of this study was to evaluate the potential for these iPSCs to differentiate into RGCs. These studies also provide an opportunity to better understand the pathogenesis of optic atrophy and to characterize a cellular model that may be useful for drug screening. It was observed that the mutant *OPA1*-derived iPSCs exhibited a significant increase in apoptosis and were not able to be differentiated into RGCs with treatment of DAPT. However, in the presence of neural induction medium, these *OPA1*^*+/−*^-iPSCs were able to be differentiated into RGCs. Noggin was identified as the critical component in this medium, and was also found to promote the differentiation of *OPA1*^*+/−*^-iPSCs into RGCs. Similarly, estrogen which was used to treat the patients with Leber’s hereditary neuropathy was found to be able to rescue *OPA1*^*+/−*^-iPSC differentiation. Under these conditions, *OPA1*^*+/−*^-iPSCs with the treatment of DAPT developed elongated axons and displayed similar morphology to that of control RGCs. Overall, these results demonstrate that an RGC differentiation protocol with DAPT treatment is able to efficiently induce the differentiation of *OPA1* mutant-derived iPSCs into RGCs in the presence of neural induction medium, Noggin, or estrogen.

## Methods

### Generation and maintenance of iPSCs

The consent forms were signed by all patients involved in this study before research initiation. The patients were subjected to a protocol that was approved by the UC Irvine and Cincinnati Children’s Medical Center IRB committee. Teratoma formation assays were undertaken with prior approval from the UC Irvine Animal Care and Use Committee (IACUC No. 2008-2855). Briefly, to obtain fibroblast cells, a needle punch biopsy of the skin was performed for each patient, and the cells were cultured as described previously [17]. Skin fibroblasts from VO (abbreviated patient’s name) and OL (abbreviated patient’s name) were then infected with a retrovirus expressing human transcription factors OCT4, SOX2, KLF4, and c-MYC, as described previously [17]. Multiple iPSC colonies were generated from each patient fibroblast cell line (VO and OL) with mutation in OPA1 (*OPA1* intron 24 c.2496 + 1 G > T, NM_015560.2), maintained and passaged in a feeder-independent culture system mTeSR1/Matrigel according to the manufacturer’s instructions (Stemcell Technologies, Vancouver, BC, Canada). After the iPSCs reached 80 % confluence, the medium was removed and the cells were rinsed once with DMEM/F12. Dispase (1 mg/mL, Life Technologies, Carlsbad, CA, USA) was added to each well of the six-well plates, and after 4–7 min incubation in 37 °C CO_2_ incubator until the colony edge started to curl off, the dispase was removed followed by two times wash with DMEM/F12 (Gibco, Carlsbad, CA, USA), then 1 ml mTeSR1 medium was added to each well of six-well plates. The cells were subsequently dissociated into small clamps with pipettes and plated on matrigel-coated plates for further expanding or maintenance. The control iPSCs were generated from skin fibroblasts of a healthy 40-year-old female using the same methods as those of VO and OL. Maintenance and subculture of the control iPSCs were using the same mTeSR1/Matrigel culture system.

### Differentiation of iPSCs

iPSCs were differentiated as described previously [16] with modification. Briefly, embryoid bodies (EBs) were formed by dissociating undifferentiated colonies with 1 mg/mL dispase and suspending the small cell aggregates in six-well ultra-low attachment plates (Nunc, Thermal Scientific, Odessa, TX, USA) in hESC medium without basic fibroblast growth factor (bFGF) (DMEM/F12, knock-out serum replacement, GlutaMax™-1, β-mercaptoethanol and Gibco MEM non-essential amino acids). On day 5, the mature EBs were then transferred to gelatin-pretreated plates and were cultured in hESC medium containing 10 % fetal bovine serum (FBS; Atlanta Biologicals, Atlanta, GA, USA). Neural rosettes appeared after several days. One week later, the neural rosettes were mechanically lifted using a syringe needle and a pipette tip, then were grown in suspension in RGC differentiation medium containing hESC medium supplemented with 10 % FBS and 10 μM DAPT (Sigma-Aldrich, St Louis, MO, USA) for 5 days to facilitate the formation of neurospheres. The neurospheres were then transferred onto poly-L-ornithine/laminin-coated plates and RGC differentiation medium containing fresh DAPT was provided every other day. On days 24, 31 or 38 following the start of differentiation, cells were fixed with 4 % paraformaldehyde (PFA) and were examined by immunofluorescence (IF) staining. For some experiments, the culture medium was supplemented with 10 % neural induction medium (Stemcell Technologies, Vancouver, BC, Canada), 100 ng/ml human recombinant Noggin (EMD Millipore, Billerica, MA, USA), or 100 nM 17β-estradiol (Sigma-Aldrich, St Louis, MO, USA).

### Immunofluorescence

IF analysis was performed as previously described [16]. Briefly, cells were fixed with 4 % PFA for 10 min at room temperature (RT), followed by two times wash with phosphate-buffered saline (PBS). After incubation for 1 hour in blocking buffer composed of 10 % goat serum (Life Technologies, Carlsbad, CA, USA) and 0.2 % Triton X-100 (Sigma-Aldrich, St Louis, MO, USA) in PBS, samples were incubated with diluted primary antibodies for 1 hour at RT. The following primary antibodies were used: rabbit anti-human TUJ1 (1:100, Fitzgerald Industrial Industries, Acton, MA, USA); mouse anti-human BRN3a (1:50, Santa Cruz Biotechnology, Dallas, Texas, USA); ISLET-1 (1:40, Developmental Studies Hybridoma Bank - University of Iowa, Iowa City, Iowa, USA); ZO-1 (1:200, Life Technologies, Carlsbad, CA, USA); and rabbit anti-human PAX6 (1:200, BioLegend, formerly Covance Antibody Products Covance, San Diego, CA, USA). The cells were then washed three times with 1× PBS, 5 min each wash, and the samples were incubated with secondary antibodies and DAPI (1:400 dilution, Life Technologies, Carlsbad, CA, USA). The secondary antibodies included goat anti-rabbit and goat anti-mouse IgGs conjugated with Alexa Fluor 488 or Alexa Fluor 594 (Life Technologies, Carlsbad, CA, USA), as appropriate. After 1 hour at RT, the cells were washed three times with 1× PBS, 5 min each wash, and visualized with a fluorescence microscope (Zeiss, Axiovert 100 M,) and analyzed with Axiovision Rel.4.8 software (Zeiss).

### Statistical analysis

For each experiment, three replicates with different colonies were performed as previously described [16]. The data are expressed as the mean ± standard deviation (SD). Student’s *t*-test was used to compare between two variables, and a *p* value <0.05 was considered statistically significant.

## Results

### Generation of iPSCs

**Fig. 1:**
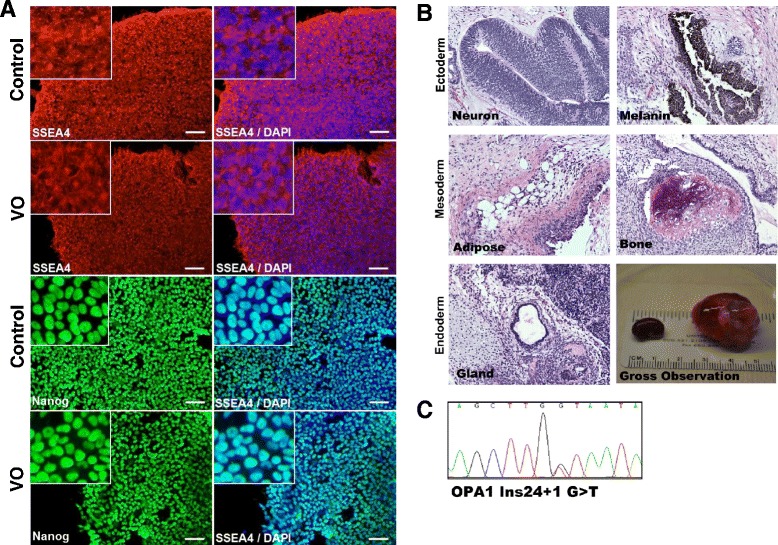
Characterization of iPSCs obtained from patient’s skin cells carrying an *OPA1* mutation. **a** Control and VO-iPSCs (patient-derived) were stained with antibodies against pluripotency cell surface markers SSEA4 (*red*) and transcriptional factor Nanog (*green*). DAPI staining of nuclei is shown in *blue*. Scale bars = 20 μM. **b** Hematoxylin and eosin staining of VO-iPSC-derived teratomas shows cell lineages derived from three germ layers, the ectoderm (neuron, skin), mesoderm (blood vessel, bone), and endoderm (gland). A gross observation of dissected teratomas is also shown (*left*, kidney capsule; *right*, testis). **c** A representative sequencing trace that confirmed the presence of an *OPA1* mutation (NM_015560.2(OPA1):c.2496 + 1G > T) in a VO-iPSC line

Fibroblasts collected from skin biopsies were cultured and differentiated in vitro. Retroviral and Sendai infections were performed to induce the exogenous expression of human transcription factors OCT, SOX2, KLF4, and c-MYC [10]. Both control and patient-derived iPSCs (VO and OL, carrying mutation NM_015560.2(OPA1):c.2496 + 1G > T) grew into colonies and exhibited a similar morphology. Immunostaining assays further demonstrated that VO-iPSCs expressed the stem cell-specific markers NANOG and SSEA-4 (Fig. 1a, data from OL-iPSCs not shown). In teratoma formation assays, the in vivo pluripotency of the derived-iPSC colonies were confirmed (Fig. 1b, data from OL-iPSCs not shown). Lastly, DNA sequencing of the VO-iPSCs proved that the original patient-specific mutation of the *OPA1* gene was maintained (Fig. 1c, data from OL-iPSCs not shown). Our results suggested that we successfully generated bona fide VO-iPSCs, which have the potential to differentiate into lineages of three germ layers.

### Mutation of *OPA1* causes increased apoptosis in *OPA1*^*+/−*^-iPSCs

**Fig. 2:**
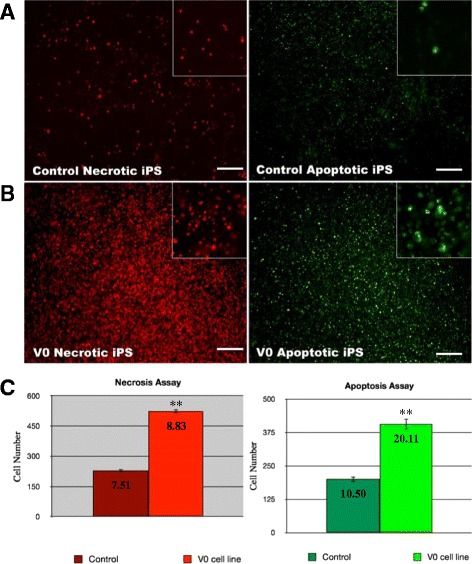
Mutation of *OPA1* increases necrosis and apoptosis in iPSCs. **a**, **b** Control and VO-iPSCs were stained with propidium iodide and YO-PRO-1 dye using an Apoptotic kit (Invitrogen) to detect necrotic cells (*red*, *left panels*) and apoptotic cells (*green*, *right panels*), respectively. Scale bars = 100 μM. **c** Each column represents the number (mean counts) of either necrotic or apoptotic cells from control and VO-iPSCs. The number of necrotic and apoptotic VO-iPSCs was significantly higher compared to that of the control iPSCs. The values shown are given as mean ± SD (*p < 0.05, **p < 0.001). SD is shown inside the boxes. *iPS* induced pluripotent stem

After the patient iPSC lines (mutant *OPA1*-iPSC) were generated and established, a large number of dead VO-iPSCs were observed during daily media changes compared to the control iPSCs. Although the function(s) of OPA1 are not fully understood, it has been shown to play an important role in mitochondrial fusion, apoptosis, ATP production, and reactive oxygen species production. Accumulating evidence further suggests that OPA1 may be a target for mitochondrial apoptotic effectors, and abnormal apoptosis leads to RGC death that is observed in DOA patients [18]. In Hela cells, downregulation of *OPA1* by RNAi led to mitochondrial fragmentation, cytochrome-c release, and apoptosis [18]. Thus, OPA1 appears to play an important role in regulating apoptosis. To investigate whether *OPA1* mutations are responsible for the increase in apoptosis observed for the *OPA1*^*+/−*^-iPSC lines, the control and VO-iPSCs were assayed for necrosis and apoptosis using an Apoptotic Kit (Invitrogen, Carlsbad, CA, USA), with the combination of YO-PRO-1 dye and propidium iodide providing a sensitive indicator for these two cell types, respectively [19]. Compared with the control iPSCs, the *OPA1*-mutant VO-iPSCs exhibited a 1.5-fold and 2-fold increase in the number of apoptotic and necrotic cells, respectively (*p* < 0.05 in each case) (Fig. 2).

### *OPA1*^*+/−*^-iPSCs do not differentiate into neural rosettes in vitro

Using a recently developed chemical stepwise protocol for the differentiation of hESCs and iPSCs into functional RGCs [16], normal control iPSCs were differentiated into neural rosettes (PAX6^+^/RX^+^) and then were cultured with the Notch inhibitor DAPT. After 3 weeks of DAPT treatment, a high percentage of cells were found to express the neuronal marker TUJ1, as well the RGC markers BRN3A, BRN3B, ATOH7, γ-Synuclein, ISLET-1, and THY-1 [16]. The differentiated RGCs additionally generated action potentials, as well as spontaneous and evoked excitatory post-synaptic currents. These data demonstrate that PAX6^+^/RX^+^ neural cells derived from hESCs and iPSCs could be differentiated into functional RGCs.

**Fig. 3:**
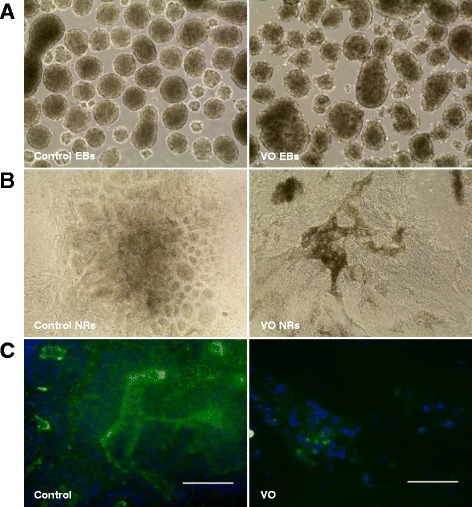
*OPA1* ^*+/−*^-iPSCs are unable to differentiate into neural rosettes (*NR*). Embryoid bodies (*EBs*) were cultured with hESC medium (without bFGF) on ultra-low attachment plates. Day 5 Ebs derived from the control iPSCs were shown in (**a**) (*left panel*). EBs were well organized and exhibited a round shape. In contrast, EBs derived from VO-iPSCs (**a**, *right panel*) exhibited irregular edges and did not have a round shape. EBs were subsequently plated onto PLO/L-coated wells and were cultured with hESC medium containing 10 % FBS. **b** Cells were imaged 5 days after EB attachment. **c** IF staining with a ZO-1 antibody (*green*) was used to show neural rosette structures. Scale bars = 20 μM

To determine whether *OPA1* mutations affect the differentiation of iPSCs into RGCs, the control, VO- and OL-iPSC aggregates were cultured with hESC medium without bFGF for 5 days. EBs derived from VO-iPSCs displayed irregular shapes and unsmoothed edges compared with the EBs derived from the control iPSCs (Fig. 3a). However, OL-EBs exhibited similar morphology to that of controls (Additional file 1: Figure S1A). Following the differentiation of these EBs (see Methods), the presence of neural rosettes was characterized with IF staining of tight junction protein ZO-1 [20]. While the control iPSCs were able to differentiate into neural rosettes that exhibited apical localization of ZO-1 (Fig. 3b and c, left panel), very few neural rosettes formed were derived from the VO- and OL-iPSCs (Fig. 3b and c, right panel; Additional file 1: Figure S1B, right panel). These results indicate that neither VO- nor OL-iPSCs can be efficiently differentiated into neural cells under these culture conditions.

### Neural rosettes derived from *OPA1*^*+/−*^-iPSCs were not able to differentiate into RGCs

**Fig. 4:**
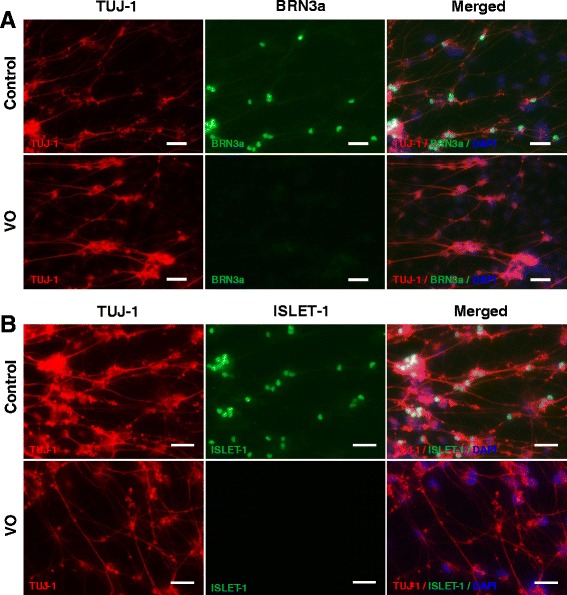
*OPA1* ^*+/−*^-iPSCs failed to differentiate into RGCs with culture medium supplemented with 10 % FBS and DAPT. Neurospheres were plated onto PLO/L-coated plates and cultured in hESC medium containing 10 μM DAPT and 10 % FBS for 7 days before fixation with 4 % PFA on day 24. Cells were stained with TUJ1 (*red*), BRN3a (*green*, **a**) and ISLET-1 (*green*, **b**) antibodies. Scale bars = 20 μM

Although the VO- and OL-iPSCs were not efficiently differentiated into neural cells, a few neural rosette-like structures were still obtained. To determine whether these VO- and OL-neural rosettes can be differentiated into RGCs, the rosettes were incubated and differentiated with the RGC differentiation medium. Putative RGCs derived from the control, VO- and OL-iPSCs were fixed with 4 % PFA on day 24 or on day 38, followed by IF staining of two RGC markers (POU-domain transcription factor BRN3a, and LIM-HD transcriptional factor ISLET-1). BRN3a is expressed in RGCs that innervate the retinal-hypothalmic/retinal-colicular pathway, and has previously been shown to be a marker for RGCs [21]. In an ISLET-1 conditional knockout mouse, a significant loss of RGCs due to apoptosis was observed, yet RGC generation was unaffected. These results suggest that ISLET-1 plays an essential role in RGC survival [22]. Previous IF analyses also detected co-localization of BRN3b and ISLET-1 in differentiating RGCs [22]. BRN3b is largely co-expressed with BRN3a in the mouse embryonic retina, and BRN3a and BRN3b have been shown to share redundant functions [23]. Therefore, BRN3a and ISLET-1 are recognized as RGC markers. In addition, TUJ-1 is a human β-tubulin III protein that is expressed in neurons, particularly in neural progenitor cells, and is widely used as a marker for neural differentiation [24, 25]. As shown in Fig. 4a and b, differentiated cells derived from the control, VO- and OL-iPSCs on day 24 were found strongly expressing TUJ-1 in both axons and body neurons. However, expression of BRN3a and ISLET-1 was only detected in differentiated neuron cells derived from the control iPSCs (Fig. 4a and b, upper panels), and not in neuron cells derived from VO- and OL-iPSCs (Fig. 4a and b, lower panels; Additional file 1: Figure S2).

To determine whether a longer cell culture period would promote *OPA1*^*+/−*^-RGC development, cells were cultured for an additional 2 weeks up to day 38. It was observed that the cells detached more readily from the bottoms of the wells, and IF analysis showed that neuron cells derived from both the control iPSCs and VO-iPSCs exhibited positive TUJ1 staining (Additional file 1: Figure S3). In contrast, expression of BRN3a and ISLET-1 was not detected in neuron cells derived from VO-iPSCs (Additional file 1: Figure S3A and B, lower panels), yet expression of these two proteins was detected in neuron cells derived from the control iPSCs (Additional file 1: Figure S3A and B, upper panels). In combination, these results suggest that VO- and OL-iPSCs are not able to differentiate into RGCs following treatment with 10 μM DAPT and 10 % FBS, thereby implying that these cells may require additional reagents or growth factors to counteract the effect(s) of *OPA1* mutations in order to gain the capacity of differentiation into RGCs.

### Neural induction medium promotes *OPA1*^*+/−*^-iPSC differentiation into RGCs

**Fig. 5:**
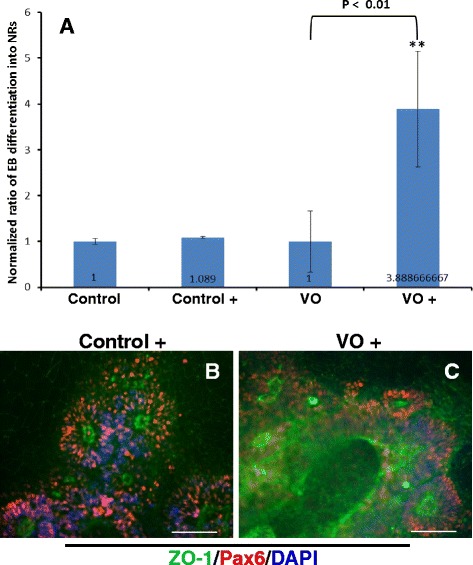
Control and VO-iPSCs were cultured in hESC medium containing 10 % FBS with and without 10 % NIM. Neural rosettes (NR) were stained with ZO-1 (*green*) and Pax6 (*red*) antibodies. In panel **a**, with (Control+ and V0+) and without (control & V0)10 % NIM Embryoid bodies (EBs) that exhibited a typical “rosette” structure were counted. Student’s t-test was used to analyze the data. All samples were normalized to the control values. *p < 0.01. Scale bars = 20 μM. Representative images are shown in panels **b** and **c**

Since VO-iPSCs were not successfully differentiated into RGCs using a previously published protocol [16], modifications of the culture conditions were tested. First, the differentiation medium was changed from hESC medium supplemented with 10 % FBS to hESC medium supplemented with both 10 % FBS and 10 % neural induction medium. As a result, the efficiency of EB differentiation of VO-iPSCs into neural rosettes increased 3.89-fold (Fig. 5a). In contrast, the efficiency of the control iPSC differentiation into neural rosettes did not significantly differ under two culture conditions (Fig. 5a). Furthermore, in an IF analysis of the newly formed neural rosettes from each group, both sets were positive for both Pax6 and ZO-1 (Fig. 5b and c).

**Fig. 6:**
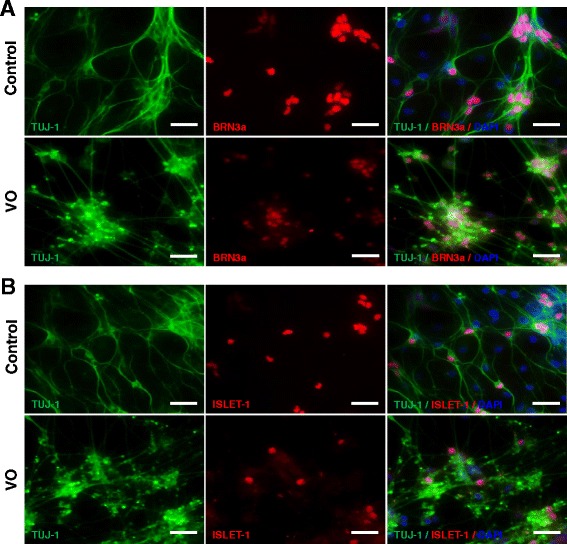
Neuron induction medium (NIM) promoted *OPA1* ^*+/−*^-RGC generation detected by IF. Neurospheres derived from the control and VO-iPSCs in the presence of NIM were plated onto PLO/L-coated plates and were cultured with hESC medium containing 10 μM DAPT, 10 % FBS, and 10 % NIM for 14 days. Cells were fixed with 4 % PFA on day 31. **a** TUJ1 (*green*) and BRN3a (*red*) staining are shown. **b** TUJ1 (*green*) and ISLET1 (*red*) staining are shown. Scale bars = 20 μM

To determine if these neural rosettes could be differentiated into RGCs, the previously published protocol was modified as follows. Briefly, mature EBs on day 6 were plated on cell culture plates coated with PLO/L, and were cultured in hESC/10 % FBS/10 % neural induction medium. After 1 week, neural rosettes were mechanically transferred to Nunc ultra-low attachment plates and were cultured as floating neuron spheres with the same differentiation medium, except 10 μM DAPT was added. After 5 days, the neurospheres were collected and plated on PLO/L-coated wells for RGC differentiation with the same DAPT-containing differentiation medium. On day 31, putative RGCs were fixed with 4 % PFA, followed by IF staining against TUJ1, BRN3a, and ISLET-1. The neural cells that derived from the VO-, OL- and control iPSCs were positive for both BRN3a and ISLET-1 (Fig. 6a and b; Additional file 1: Figure S4). Next, the efficiency of RGC differentiation was quantified using the ratio of RGC-positive signal versus DAPT signal (Additional file 1: Figure S5). The differentiation efficiency between VO-iPSCs and the control iPSCs to RGCs was found to be very similar (*p* = 0.076 and *p* = 0.084 for ISLET-1 and BRN3a, respectively).

### Noggin promotes the differentiation of *OPA1*^*+/−*^-iPSCs into neural stem cells

**Fig. 7:**
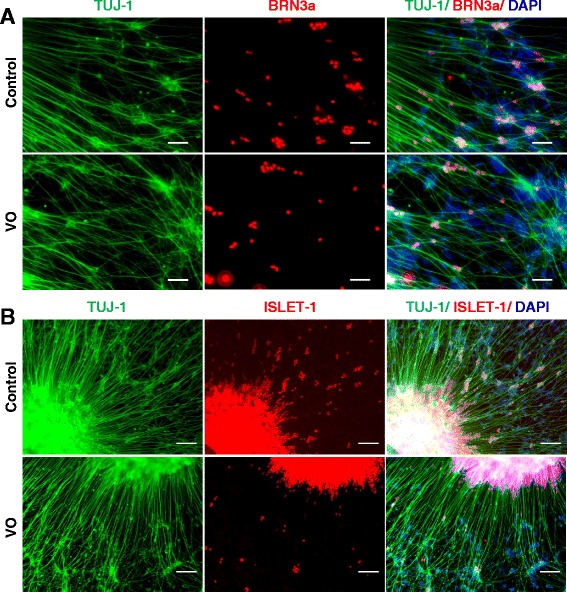
Noggin rescued *OPA1* ^*+/−*^-iPSC differentiation into RGCs confirmed by IF. Mature EBs were cultured with 100 ng/mL Noggin to obtain putative RGCs. Cells were fixed with 4 % PFA on day 31. **a** TUJ-1 (*green*) and BRN3a (*red*) staining are shown. **b** TUJ-1 (*green*) and ISLET1 (*red*) staining are shown. Scale bars = 50 μM in panel (**a**) and 100 μM in panel (**b**)

Noggin is a secreted signaling molecule that plays an important role in regulating several signaling pathways during animal development. In *Xenopus*, Noggin-treated pluripotent cells were found to be able to differentiate into a multipotent retinal progenitor lineage, which facilitated functional eye formation following transplantation in vivo [26]. In 2009, Lan and colleagues also reported that a high-dose treatment of Noggin promoted retinal cell differentiation in *Xenopus* embryonic stem cells [27]. During the differentiation of mouse iPSCs, the addition of Noggin and fibroblast growth factor 2 into the differentiation medium for a short period of time resulted in a significant enhancement of retinal progenitor cell growth [28]. To evaluate the potential of Noggin to induce the differentiation of human iPSCs into neural retinal cells, especially patient-derived iPSCs containing mutations, a revised protocol was tested. Briefly, mature EBs on day 6 were plated on laminin-coated plates and cultured in hESC medium containing 10 % FBS and 100 ng/mL Noggin. After 7 days, the cells were cultured as floating neural spheres in RGC differentiation medium containing hESC medium, 10 % FBS, 10uM DAPT, and 100 ng/mL Noggin. After 5 days, the neurospheres were plated on PLO/L-coated plates and differentiated for another 14 days. On day 31, putative RGCs were fixed in 4 % PFA and IF analysis detected robust expression of BRN3a and ISLET-1 in the neuron cells derived from VO- and OL-iPSCs (Fig. 7a and b; Additional file 1: Figure S6). Similar expression patterns were observed for the RGCs derived from the control iPSCs (Fig. 7a and b).

**Fig. 8:**
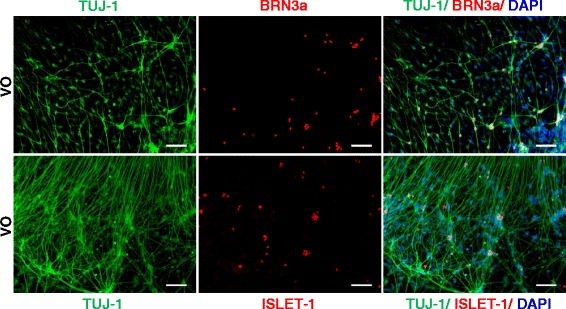
Noggin treatment only during *OPA1* ^*+/−*^-iPSC differentiation into neural rosettes could rescue *OPA1* ^*+/−*^-RGC generation. Control and VO-iPSCs were incubated with 100 ng/mL Noggin in the cell culture medium only during the differentiation of neural rosettes and Noggin was removed in afterward differentation. Cells were fixed with 4 % PFA on day 31. *Upper panel*, TUJ1 (*green*) and BRN3a (*red*) staining are shown. *Lower panel*, TUJ1 (*green*) and ISLET-1 (*red*) staining are shown. Scale bars = 100 μM. Data from the control under the same treatment are not shown

To examine the function of Noggin during RGC differentiation, two separate, yet parallel, experiments were performed at the same time. In the first experiment, iPSCs were treated with Noggin during the differentiation stage of neural rosettes. However, the subsequent culture steps did not include Noggin. In the second experiment, Noggin was present in the RGC differentiation medium and was not present in the medium used to culture the neural rosettes. VO neuron cells in the first experiment exhibited robust expression of BRN3a and ISLET-1 (Fig. 8). However, in the second experiment, very few neural rosettes were available for differentiation, and IF staining showed very sparse expression of BRN3a and ISLET-1 (data not shown). Based on the results of these two experiments, it appears that Noggin promotes the formation of neural stem cells (neural rosettes), and does not induce RGC differentiation during the process of VO-iPSC differentiation.

### 17β-estradiol promotes *OPA1*^*+/−*^-iPSC differentiation into RGCs

**Fig. 9:**
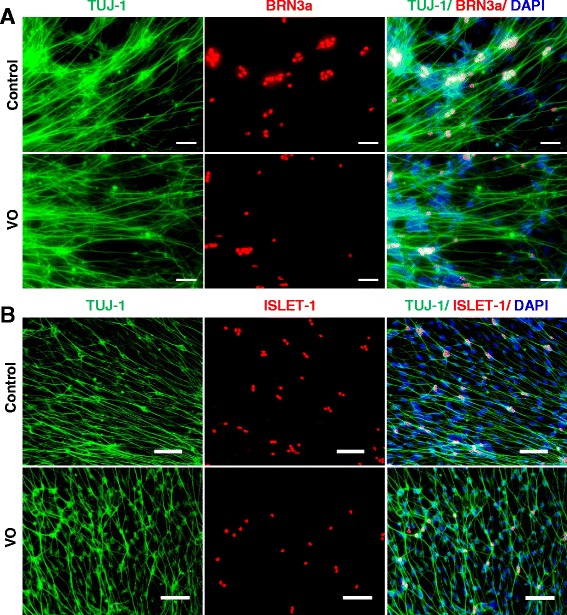
Addition of 17β-estradiol in RGC differentiation medium promoted generation of *OPA1* ^*+/−*^-RGCs. Control and VO RGCs were incubated with 100 nM 17β-estradiol in the cell culture medium during all of the differentiation stages, including iPSC culturing. Putative RGCs were then fixed with 4 % PFA on day 31. **a** TUJ1 (*green*) and BRN3a (*red*) staining are shown. **b** TUJ1 (*green*) and ISLET-1 (*red*) staining are shown. Scale bars = 50 μM in panel (**a**) and 100 μM in panel (**b**)

In a cybrid cell model of Leber’s hereditary optic neuropathy (LHON) involving the selective loss of RGCs, treatment with 17β-estradiol was found to significantly decrease levels of apoptosis, to increase cell viability, and to enhance mitochondrial biogenesis [29]. In addition, immune-peroxidase staining confirmed that estrogen receptor-β is located on the mitochondria of human RGCs, thereby implying that estrogen mediates its function via this receptor [29]. Since VO-iPSCs exhibited significant apoptosis, and 17β-estradiol has proved to be an inhibitor of apoptosis, the cell culture medium used for iPSC differentiation was supplemented with 100 nM 17β-estradiol. When VO- and OL-iPSCs were stained with antibodies specific for detection of BRN3a and ISLET-1, both proteins were found to be highly expressed by neuron cells derived from VO- and OL-iPSCs on day 31 (Fig. 9; Additional file 1: Figure S7), similar to the RGCs derived from the control iPSCs (data not shown). Taken together, these results indicate that 17β-estradiol can promote RGC development possibly through inhibition of apoptosis, and thus may serve as a potential therapeutic agent for *OPA1* mutation-related optic atrophy.

## Discussion

RGC loss is critical for the development of optic atrophy, LHON, and glaucoma [30–32]. Moreover, there is currently no effective treatment for this group of diseases. Therefore, cell replacement may represent a potential therapy for lost neurons [20, 33]. Although hESCs have been used to generate neural retinal precursors [34], there are ethical considerations associated with this approach, as well as the potential for immune rejection [35]. iPSC technology circumvents these issues since patient-specific iPSCs can be generated, and these iPSCs have the ability to differentiate into various cell types [23, 36, 37].

It has also been reported recently that retinal ganglion-like cells can be generated from iPSCs [28, 38–40]. Several protocols have been developed and published for the retina-specific differentiation of stem cells. These protocols employ non-adherent culturing of cells to induce neurosphere formation, and the cells are subsequently differentiated into retinal progenitor epithelial cells and neural epithelial rosette-containing colonies. Using this system, early retinal development from hESCs and iPSCs has been modeled [41].

To promote the differentiation of retinal progenitor cells into ganglion cells, we recently developed a protocol that included the Notch antagonist DAPT [16]. Using this rather straightforward protocol, lineage differentiation to obtain RGCs was significantly enhanced. This result also suggests that Notch signaling plays an important role in stem cell differentiation, and may have a clinical application. When iPSCs harboring *OPA1* mutations were evaluated, increased apoptosis was observed. However, in the presence of neural induction medium, these iPSCs were still able to differentiate into RGCs. Taken together, these results suggest that the primary pathogenesis of optic atrophy due to an *OPA1* mutation is most likely due to a reduced number of RGCs, secondary to increased levels of RGC apoptosis.

Noggin is the major component of neural induction medium, and as a potent inhibitor of BMP enhances eye field differentiation [42]. In transgenic mice overexpressing *Noggin*, apoptosis of the developing eyelid epithelium was found to be inhibited via the BMP-Smad pathway [43]. IF analysis and in situ hybridization of proteins and receptors involved in the BMP pathway also confirmed these results. It has been shown in a hippocampal neurodegeneration animal model that neuropeptide Y promotes neuroprotection and enhances endogenous neurogenesis by enhancing expression of *Noggin* [44]. These findings correlate with our results showing that Noggin played an important role in promoting generation of neuron stem cells, and thus led to a success in *OPA1*^*+/−*^-iPSC differentiation into RGCs (Fig. 7a and b and Fig. 8). Our results from two separate, yet parallel experiments showed that Noggin significantly enhanced neuron rosette formation, and thus promoted *OPA1*^*+/−*^-RGC generation. However, the detailed molecular mechanism by which Noggin affects *OPA1*^*+/−*^-RGC differentiation is yet to be elucidated. The effectiveness of Noggin was replicable. Without adding Noggin in the differentiation medium, very few neuron rosettes formed and were not able to further differentiate into RGCs. *Noggin* mutations in mouse embryos have been associated with a significant increase in neural crest cell apoptosis [45], indicating that Noggin plays an important role in regulating apoptosis during neural crest development. Based on the results of the present study, we further hypothesize that Noggin can partially reverse the differentiation defects mediated by *OPA1* mutations.

17β-estradiol is a potent steroid hormone and plays an important protective role in many organ systems in addition to the reproductive system. 17β-estradiol has also been shown to function as a neuromodulator and neuroprotectant by manipulating various molecular pathways [46]. For example, using a hippocampal slice culture model, treatment with estrogen in vitro has been shown to inactivate the cell death mediator, GSK-3β, via phosphorylation [47]. As a result, neuron viability and survival were promoted, and estrogen treatment was identified as a promising approach for preventing cell death. In an in vivo mouse model of glaucoma, a significant loss of RGCs was observed [48]. However, treatment with 17β-estradiol was found to prevent RGC apoptosis, and RGC neurofiber integrity was protected [48]. Given that estrogen receptor-β is located in the ganglion cell layer [49], it is very likely that estrogen plays an important role in preventing RGC loss and promoting RGC development through interaction with estrogen receptor-β. Accordingly, the present results show that RGCs were generated from VO- and OL-iPSCs after addition of estrogen into the RGC differentiation medium. Thus, it appears that estrogen can counteract the effects mediated by mutated OPA1 proteins in terms of apoptosis inhibition.

The molecular mechanism(s) by which *OPA1* mutations lead to increased apoptosis are not completely understood, and remain controversial. It has been shown that inhibition of the mitochondrial fission machinery and upregulation of mitochondrial fusion can both regulate cell death, and OPA1 is an important regulator of mitochondrial fusion. However, OPA1 protects cells from apoptosis by preventing cytochrome-c release, which can occur independently from mitochondrial fusion. OPA1 also controls the shape of mitochondrial cristae by maintaining tight junctions during apoptosis that is induced by oligomerization of OPA1. Conversely, BCL-2 can widen cristae junctions and disrupt OPA1 oligomers. Thus, the anti-apoptosis function of OPA1 is distinct from its role in mitochondrial fusion, yet is not distinct from its role in cristae remodeling [50]. It is anticipated that further study of this dynamic protein may identify additional novel therapies as well.

## Conclusions

Recently, we have developed a straightforward protocol to differentiate iPSCs into functional RGCs in which neural rosettes were cultured in the presence of the Notch inhibitor DAPT. Using this protocol, here we show that *OPA1* mutations cause a significant increase in apoptosis in iPSCs, and iPSCs harboring *OPA1* mutation were unable to differentiate into RGCs. However, both noggin and β-estrogen can rescue this phenotype, suggesting apoptosis mediated by *OPA1* mutations plays an important role in the pathogenesis of optic atrophy, and noggin and β-estrogen could be potential therapeutic agents. These results will further our understanding of how *OPA1* mutations lead to RGC loss and optic atrophy.

## Additional file

Additional file 1: Figure S1.
*OPA1* ^*+/−*^-iPSCs (OL) are unfavorable to differentiate into neural rosette. Culture conditions were the same as those in Fig. 3. Panel A shows day 5 EBs, which were derived from control and OL-iPSCs, respectively. Morphologically, OL-EBs look similar to control EBs. Panel B shows neuron rosettes (NRs) after 5 days of EB attachment. Fewer and smaller neuron rosettes were derived from OL-EBs, compared with neuron rosettes from control EBs. Figure S2 *OPA1* ^*+/−*^-iPSCs (OL) failed to differentiate into RGCs with culture medium supplemented with 10 % FBS and DAPT. Differentiation method in Fig. 4 was followed. Putative RGCs were fixed on day 24 before fixation, followed by IF staining. Upper panel shows staining of TUJ1 (green) and BRN3a (red), while lower panel displays staining of TUJ1 (green) and ISLET1 (red). The scale bars equal 50 μM. Figure S3 IF analysis of RGCs derived from the control and VO-iPSCs on day 38. Neurospheres were plated onto PLO/L-coated plates and cultured in hESC medium containing 10 μM DAPT and 10 % FBS for 21 days before fixation with 4 % PFA on day 38. Cells were stained with TUJ1 (green), BRN3a (red) and ISLET-1 (red) antibodies. The scale bars equal 20 μM. Figure S4 NIM promoted *OPA1* ^*+/−*^-RGC (OL) generation detected by IF. Putative OL-RGCs derived from OL-iPSCs were cultured with hESC medium containing 10 μM DAPT, 10 % FBS, and 10 % neural induction medium (NIM) for 14 days before fixation on day 31. Upper panel shows TUJ1 (green) and BRN3a (red) staining. Lower panel shows staining of TUJ1 (green) and ISLET1 (red). The scale bars equal 50 μM. Figure S5 Quantification of RGC differentiation efficiency. The culture medium used for RGC differentiation contained 10 % neural induction medium. Samples were normalized to the control BRN3a/DAPI staining or the control ISLET-1/DAPI staining. The number of BRN3a or ISLET-1 signals versus the number of DAPI signals was calculated. A *p-*value of 0.084 was obtained for the BRN3a signal comparison between the control and VO, and a *p-*value of 0.076 was obtained for the ISLET-1 signal comparison between the control and VO. Student’s *t*-test was used to analyze differences between two groups. Figure S6 Noggin rescued *OPA1* ^*+/−*^-iPSC (OL) differentiation into RGCs confirmed by IF. Mature EBs were cultured with hES medium containing 10 % FBS and 100 ng/mL Noggin to obtain neuron rosettes, followed by culturing putative RGCs with hES medium containing 10 % FBS, DAPT and 100 ng/mL Noggin. Cells were fixed with 4 % PFA on day 31. Upper panel shows IF staining of TUJ-1 (green) and BRN3a (red), whereas lower panel shows staining of TUJ-1 (green) and ISLET1 (red). The scale bars equal 50 μM in both panels. Figure S7 Addition of 17β-estradiol in RGC differentiation medium promoted generation of *OPA1* ^*+/−*^-RGCs (OL). Putative RGCs were incubated with 100 nM 17β-estradiol in the cell culture medium during all of the differentiation stages, followed by fixation on day 31. Upper panel shows IF staining against TUJ1 (green) and BRN3a (red). Lower panel shows TUJ1 (green) and ISLET-1 (red) staining. The scale bars equal 50 μM. (PPTX 12904 kb)

## Abbreviations

bFBF: Basic fibroblast growth factor
DAPT: N-[N-(3,5-difluorophenacetyl)-l-alanyl]-S-phenylglycine t-butyl ester
DOA: Autosomal dominant optic atrophy
EB: Embryoid body
FBS: Fetal bovine serum
hESC: Human embryonic stem cell
IF: Immunofluorescence iPSCs, Induced pluripotent stem cells
LHON: Leber’s hereditary optic neuropathy
OPA1: Optic atrophy 1
PBS: Phosphate-buffered saline
PFA: Paraformaldehyde
RGC: Retinal ganglion cell
RT: Room temperature
