# Supplementary material for: Modeling autosomal dominant optic atrophy using induced pluripotent stem cells and identifying potential therapeutic targets
Source: Stem Cell Res Ther. 2016 Jan 7;7:2. doi: 10.1186/s13287-015-0264-1 (PMC4704249; doi:10.1186/s13287-015-0264-1)
Supplement: Additional file 1: Figure S1. — OPA1 +/−-iPSCs (OL) are unfavorable to differentiate into neural rosette. Culture conditions were the same as those in Fig. 3. Panel A shows day 5 EBs, which were derived from control and OL-iPSCs, respectively. Morphologically, OL-EBs look similar to control EBs. Panel B shows neuron rosettes (NRs) after 5 days of EB attachment. Fewer and smaller neuron rosettes were derived from OL-EBs, compared with neuron rosettes from control EBs. Figure S2 OPA1 +/−-iPSCs (OL) failed to differentiate into RGCs with culture medium supplemented with 10 % FBS and DAPT. Differentiation method in Fig. 4 was followed. Putative RGCs were fixed on day 24 before fixation, followed by IF staining. Upper panel shows staining of TUJ1 (green) and BRN3a (red), while lower panel displays staining of TUJ1 (green) and ISLET1 (red). The scale bars equal 50 μM. Figure S3 IF analysis of RGCs derived from the control and VO-iPSCs on day 38. Neurospheres were plated onto PLO/L-coated plates and cultured in hESC medium containing 10 μM DAPT and 10 % FBS for 21 days before fixation with 4 % PFA on day 38. Cells were stained with TUJ1 (green), BRN3a (red) and ISLET-1 (red) antibodies. The scale bars equal 20 μM. Figure S4 NIM promoted OPA1 +/−-RGC (OL) generation detected by IF. Putative OL-RGCs derived from OL-iPSCs were cultured with hESC medium containing 10 μM DAPT, 10 % FBS, and 10 % neural induction medium (NIM) for 14 days before fixation on day 31. Upper panel shows TUJ1 (green) and BRN3a (red) staining. Lower panel shows staining of TUJ1 (green) and ISLET1 (red). The scale bars equal 50 μM. Figure S5 Quantification of RGC differentiation efficiency. The culture medium used for RGC differentiation contained 10 % neural induction medium. Samples were normalized to the control BRN3a/DAPI staining or the control ISLET-1/DAPI staining. The number of BRN3a or ISLET-1 signals versus the number of DAPI signals was calculated. A p-value of 0.084 was obtained for the BRN3a signal comparison between the [file 13287_2015_264_MOESM1_ESM.pptx]

## Slide 1
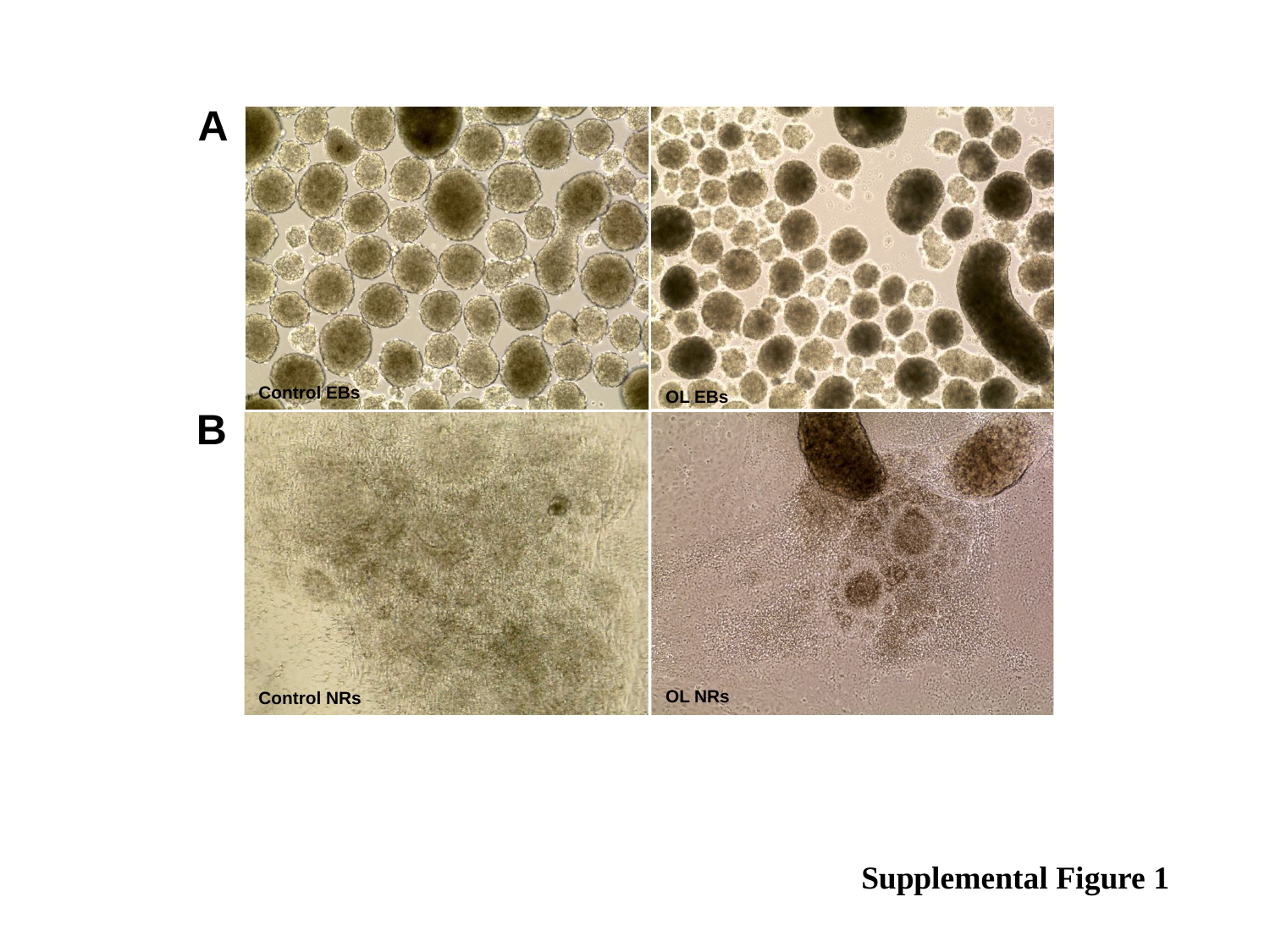

A
Control EBs
OL EBs
B
OL NRs
Control NRs
Supplemental Figure 1

## Slide 2
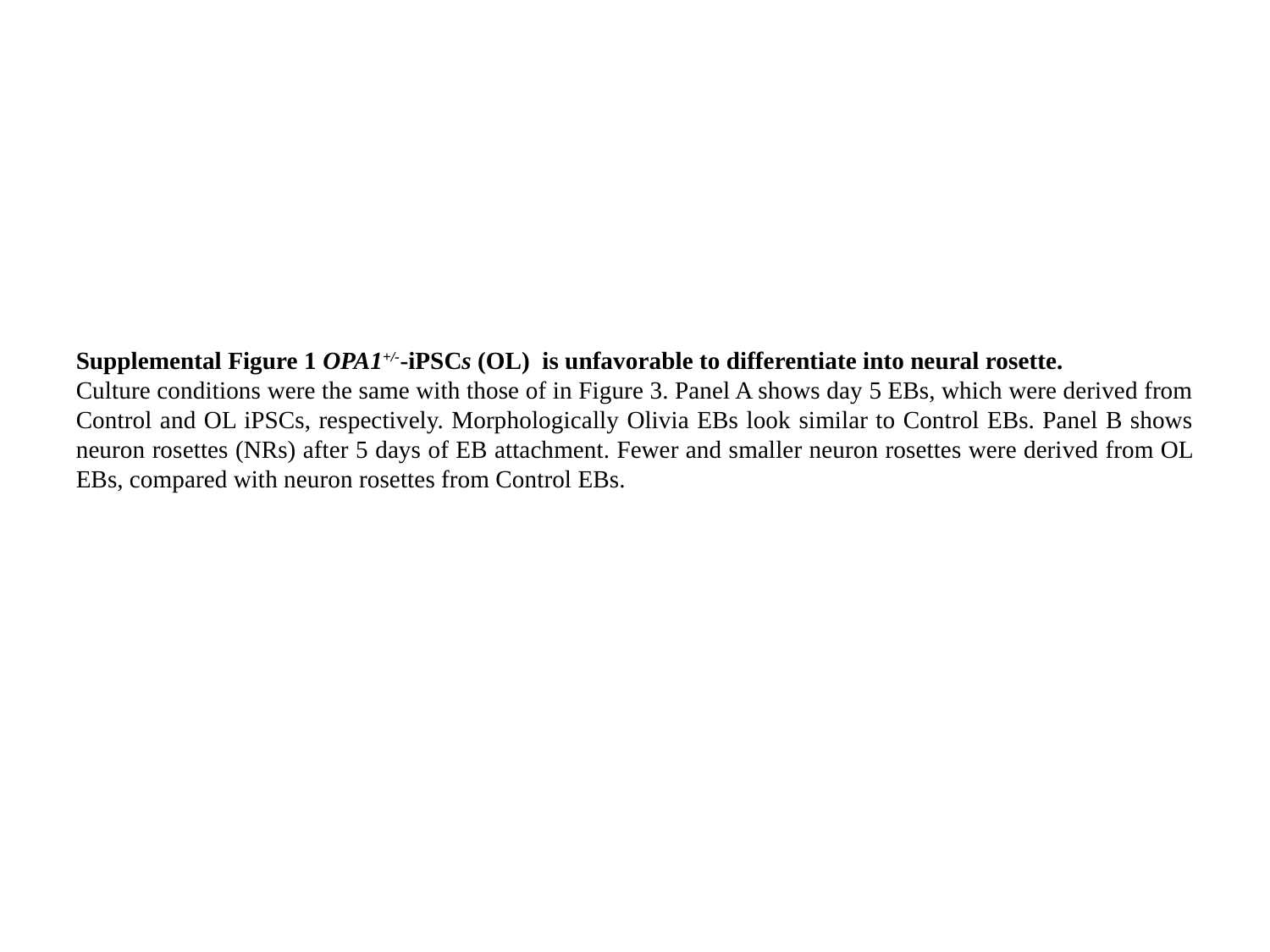

Supplemental Figure 1 OPA1+/--iPSCs (OL) is unfavorable to differentiate into neural rosette.
Culture conditions were the same with those of in Figure 3. Panel A shows day 5 EBs, which were derived from Control and OL iPSCs, respectively. Morphologically Olivia EBs look similar to Control EBs. Panel B shows neuron rosettes (NRs) after 5 days of EB attachment. Fewer and smaller neuron rosettes were derived from OL EBs, compared with neuron rosettes from Control EBs.

## Slide 3
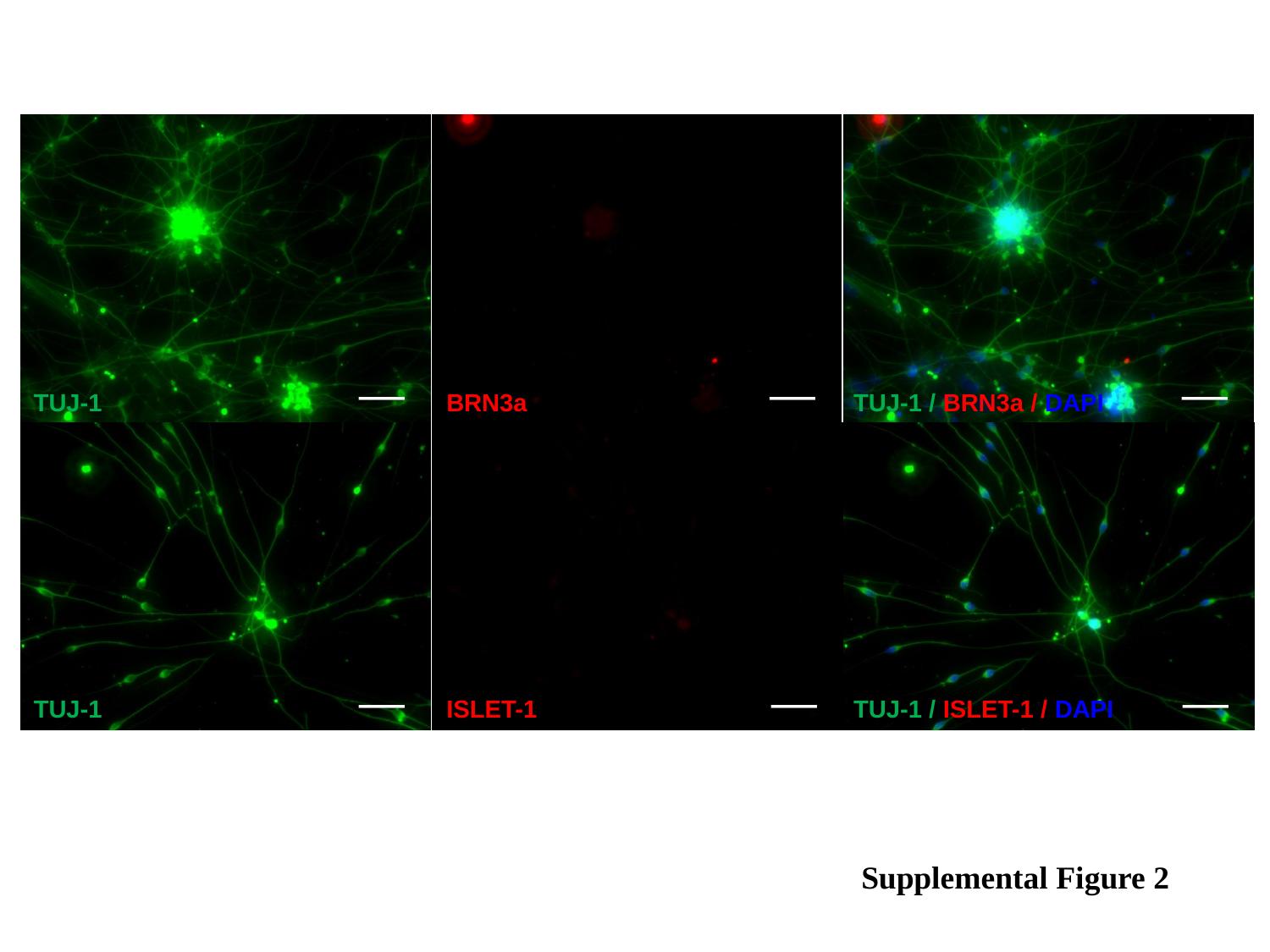

TUJ-1
BRN3a
TUJ-1 / BRN3a / DAPI
TUJ-1
ISLET-1
TUJ-1 / ISLET-1 / DAPI
Supplemental Figure 2

## Slide 4
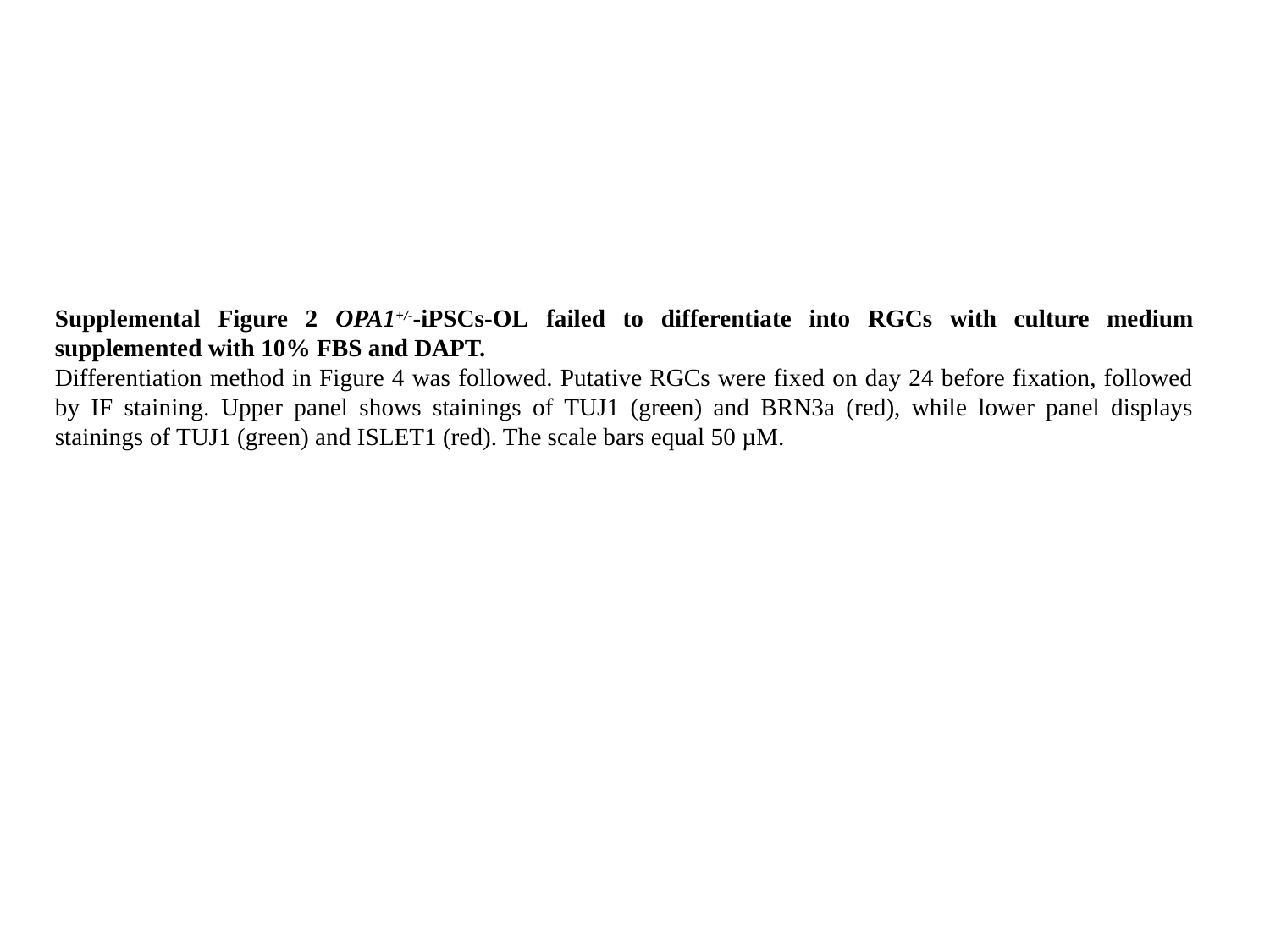

Supplemental Figure 2 OPA1+/--iPSCs-OL failed to differentiate into RGCs with culture medium supplemented with 10% FBS and DAPT.
Differentiation method in Figure 4 was followed. Putative RGCs were fixed on day 24 before fixation, followed by IF staining. Upper panel shows stainings of TUJ1 (green) and BRN3a (red), while lower panel displays stainings of TUJ1 (green) and ISLET1 (red). The scale bars equal 50 µM.

## Slide 5
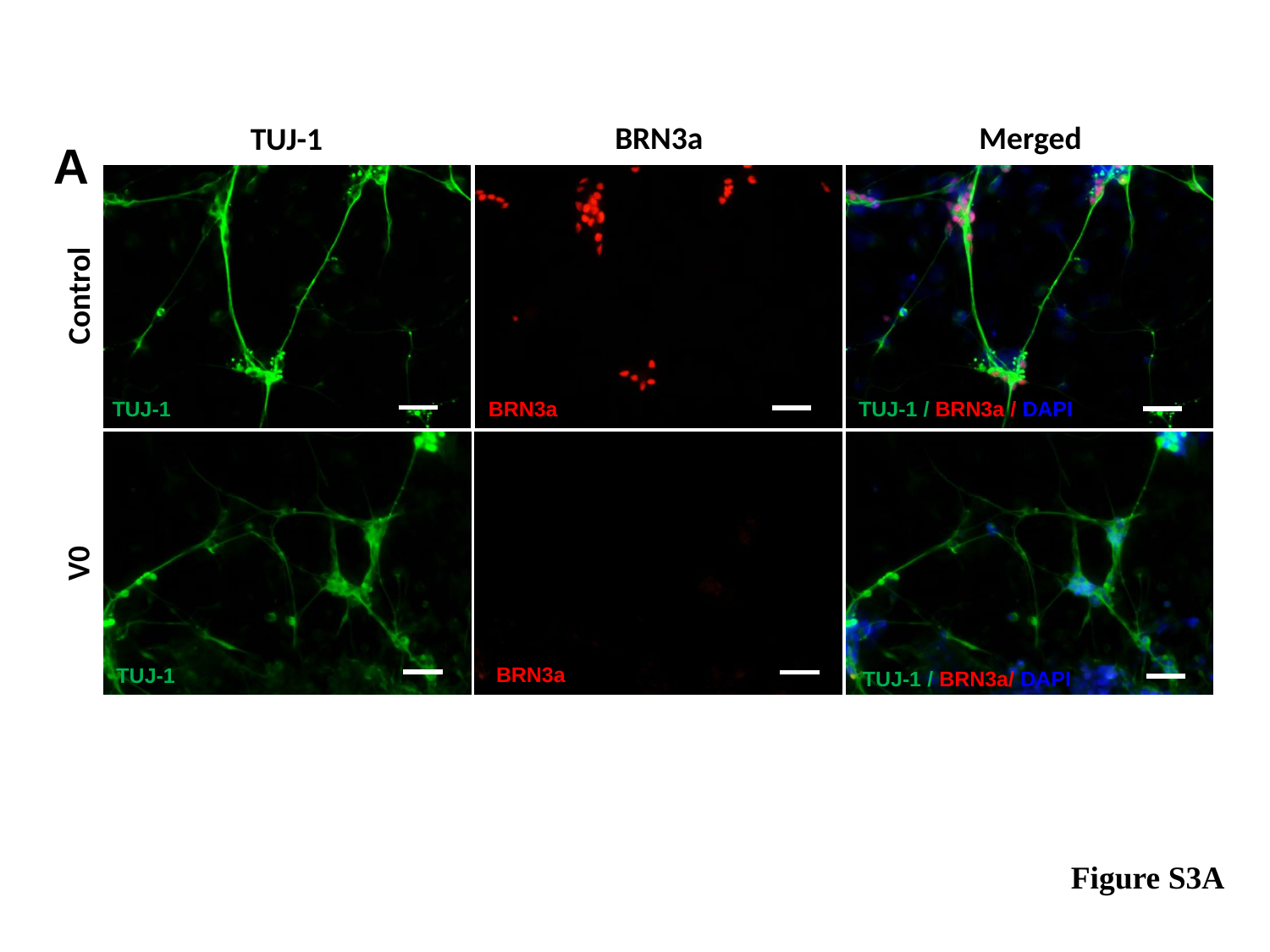

BRN3a
Merged
TUJ-1
A
BRN3a
TUJ-1
TUJ-1 / BRN3a / DAPI
Control
TUJ-1
BRN3a
TUJ-1 / BRN3a/ DAPI
V0
Figure S3A

## Slide 6
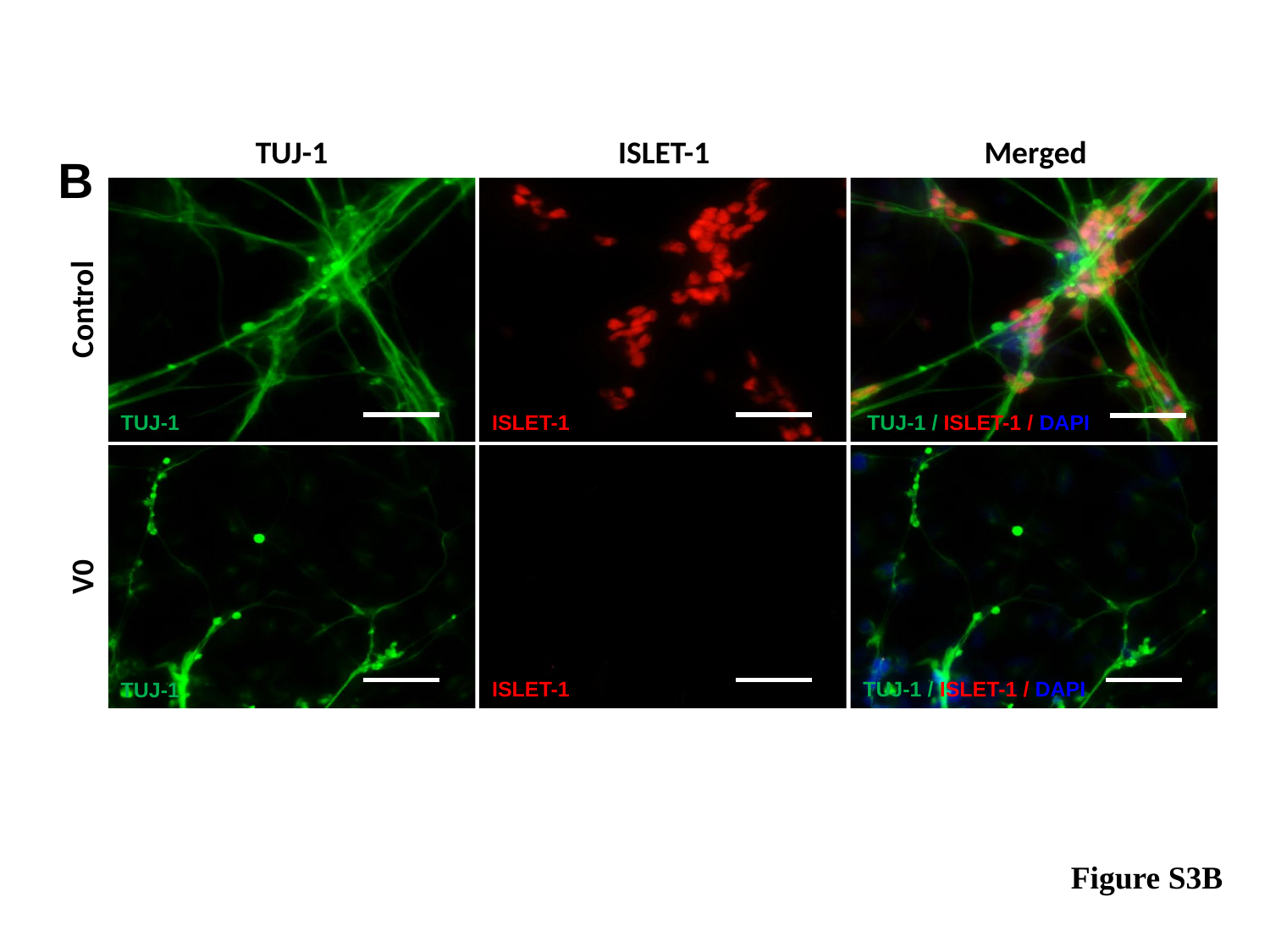

TUJ-1
ISLET-1
Merged
TUJ-1 / ISLET-1 / DAPI
TUJ-1
ISLET-1
Control
TUJ-1
ISLET-1
TUJ-1 / ISLET-1 / DAPI
V0
B
Figure S3B

## Slide 7
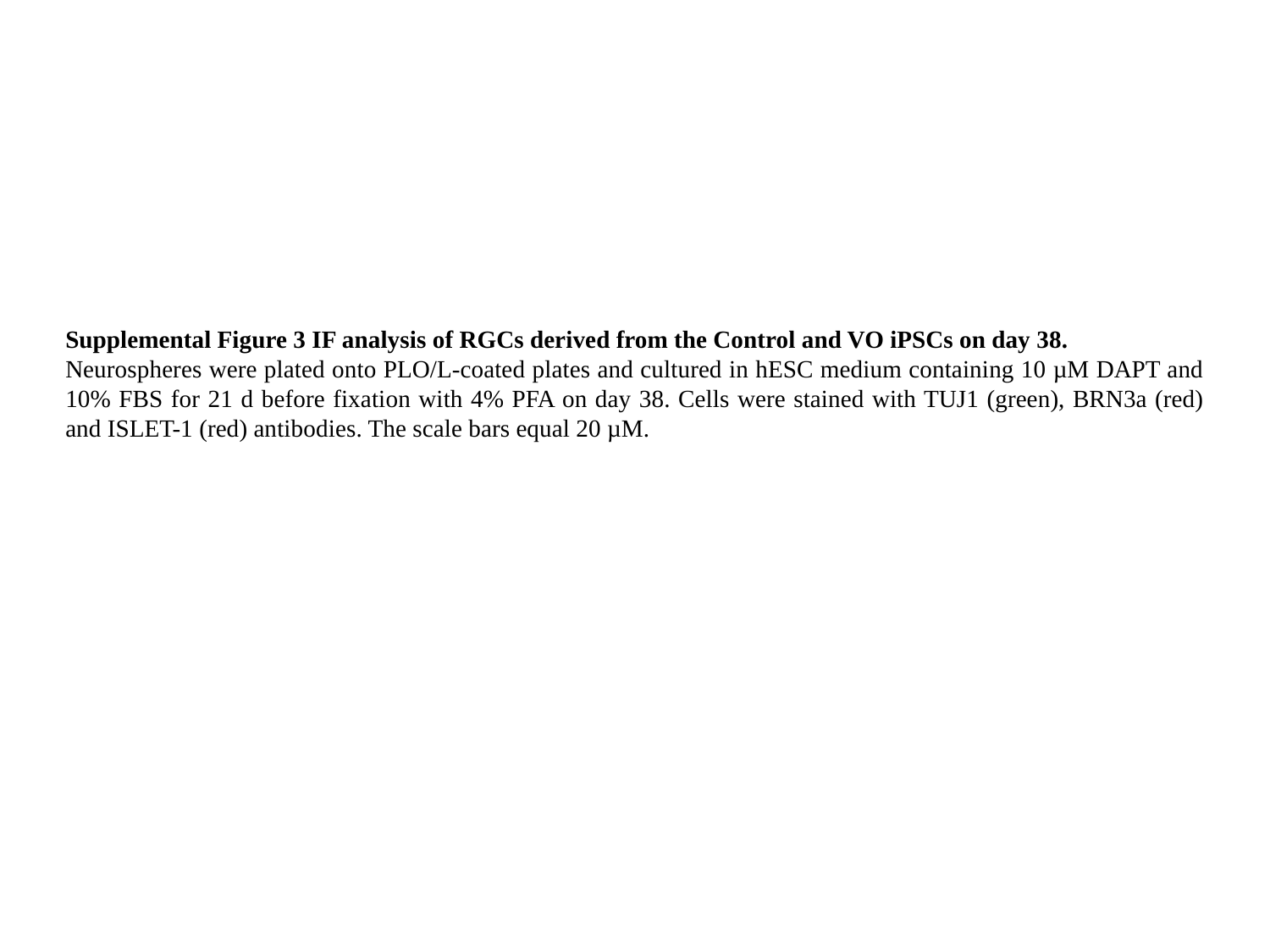

Supplemental Figure 3 IF analysis of RGCs derived from the Control and VO iPSCs on day 38.
Neurospheres were plated onto PLO/L-coated plates and cultured in hESC medium containing 10 µM DAPT and 10% FBS for 21 d before fixation with 4% PFA on day 38. Cells were stained with TUJ1 (green), BRN3a (red) and ISLET-1 (red) antibodies. The scale bars equal 20 µM.

## Slide 8
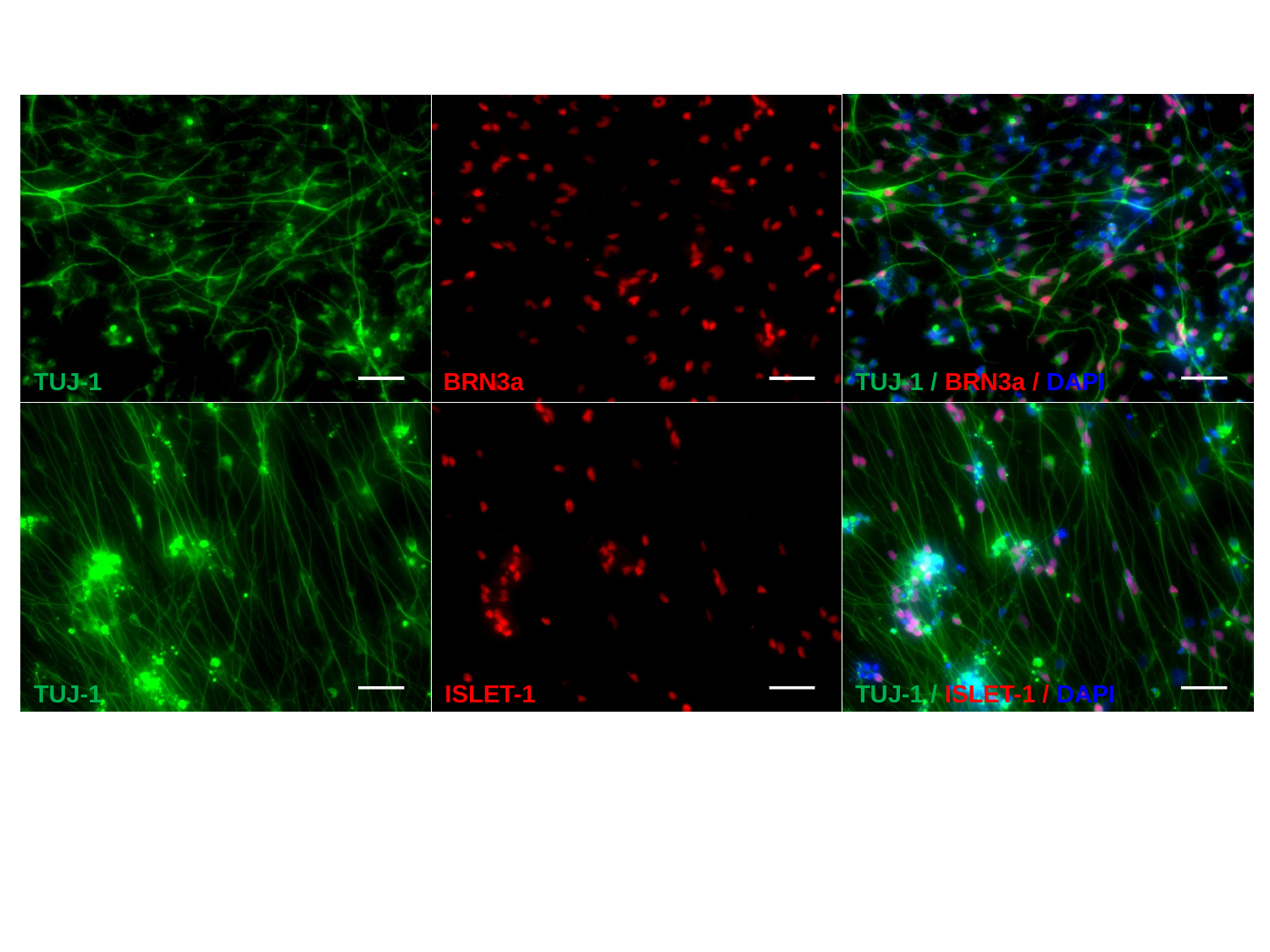

TUJ-1
BRN3a
TUJ-1 / BRN3a / DAPI
TUJ-1
ISLET-1
TUJ-1 / ISLET-1 / DAPI

## Slide 9
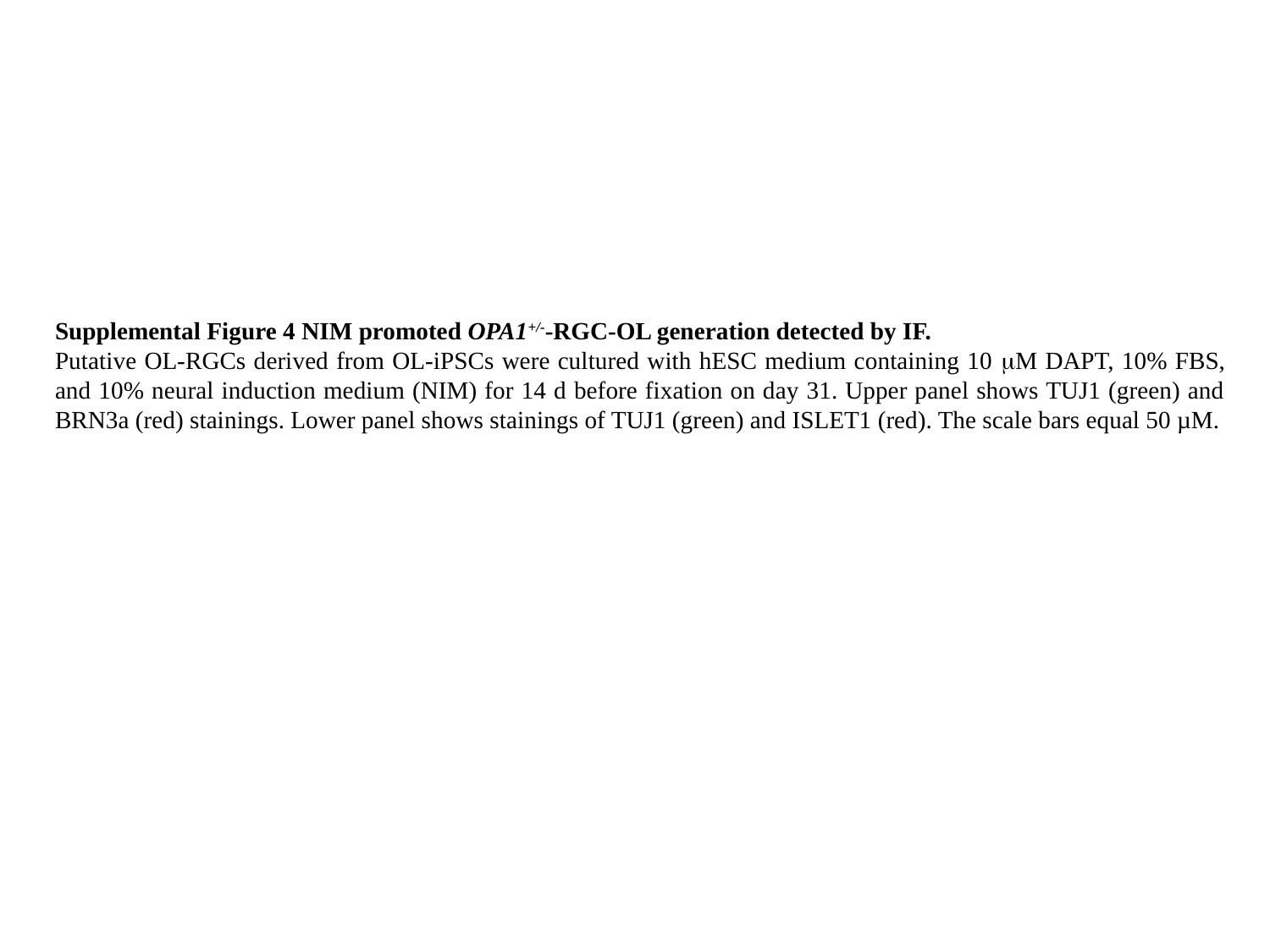

Supplemental Figure 4 NIM promoted OPA1+/--RGC-OL generation detected by IF.
Putative OL-RGCs derived from OL-iPSCs were cultured with hESC medium containing 10 mM DAPT, 10% FBS, and 10% neural induction medium (NIM) for 14 d before fixation on day 31. Upper panel shows TUJ1 (green) and BRN3a (red) stainings. Lower panel shows stainings of TUJ1 (green) and ISLET1 (red). The scale bars equal 50 µM.

## Slide 10
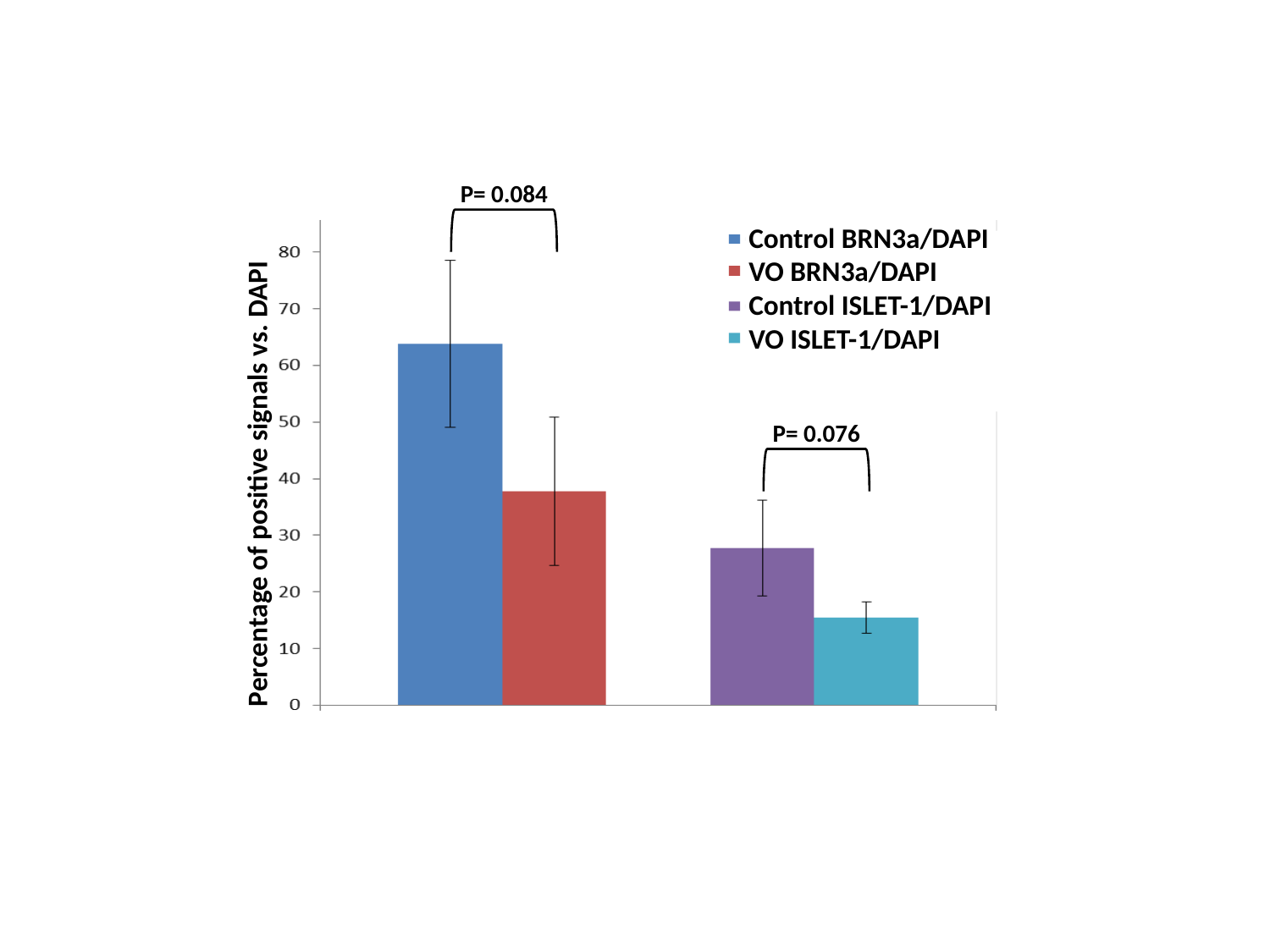

P= 0.084
Percentage of positive signals vs. DAPI
Control BRN3a/DAPI
VO BRN3a/DAPI
Control ISLET-1/DAPI
VO ISLET-1/DAPI
P= 0.076

## Slide 11
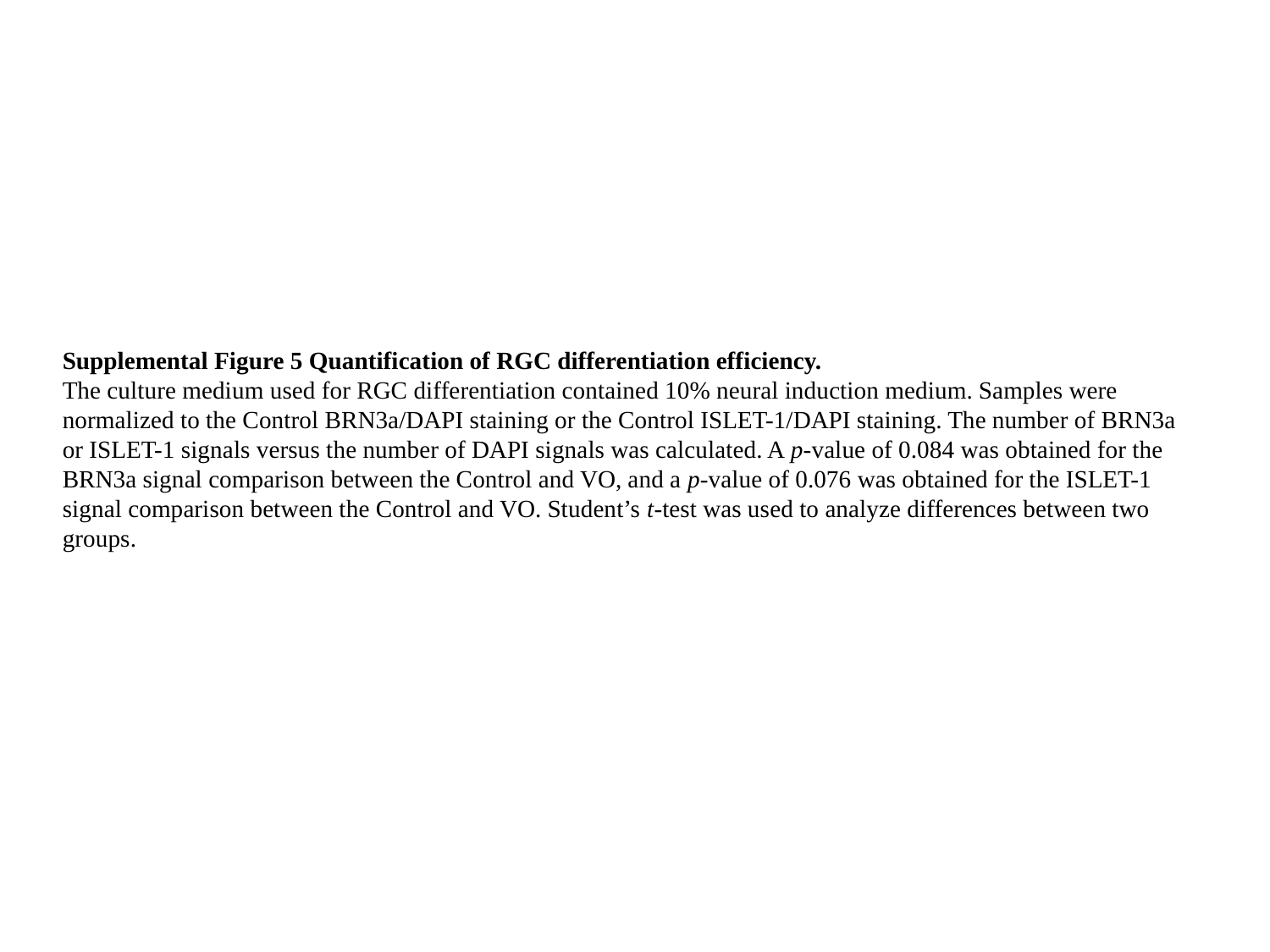

Supplemental Figure 5 Quantification of RGC differentiation efficiency.
The culture medium used for RGC differentiation contained 10% neural induction medium. Samples were normalized to the Control BRN3a/DAPI staining or the Control ISLET-1/DAPI staining. The number of BRN3a or ISLET-1 signals versus the number of DAPI signals was calculated. A p-value of 0.084 was obtained for the BRN3a signal comparison between the Control and VO, and a p-value of 0.076 was obtained for the ISLET-1 signal comparison between the Control and VO. Student’s t-test was used to analyze differences between two groups.

## Slide 12
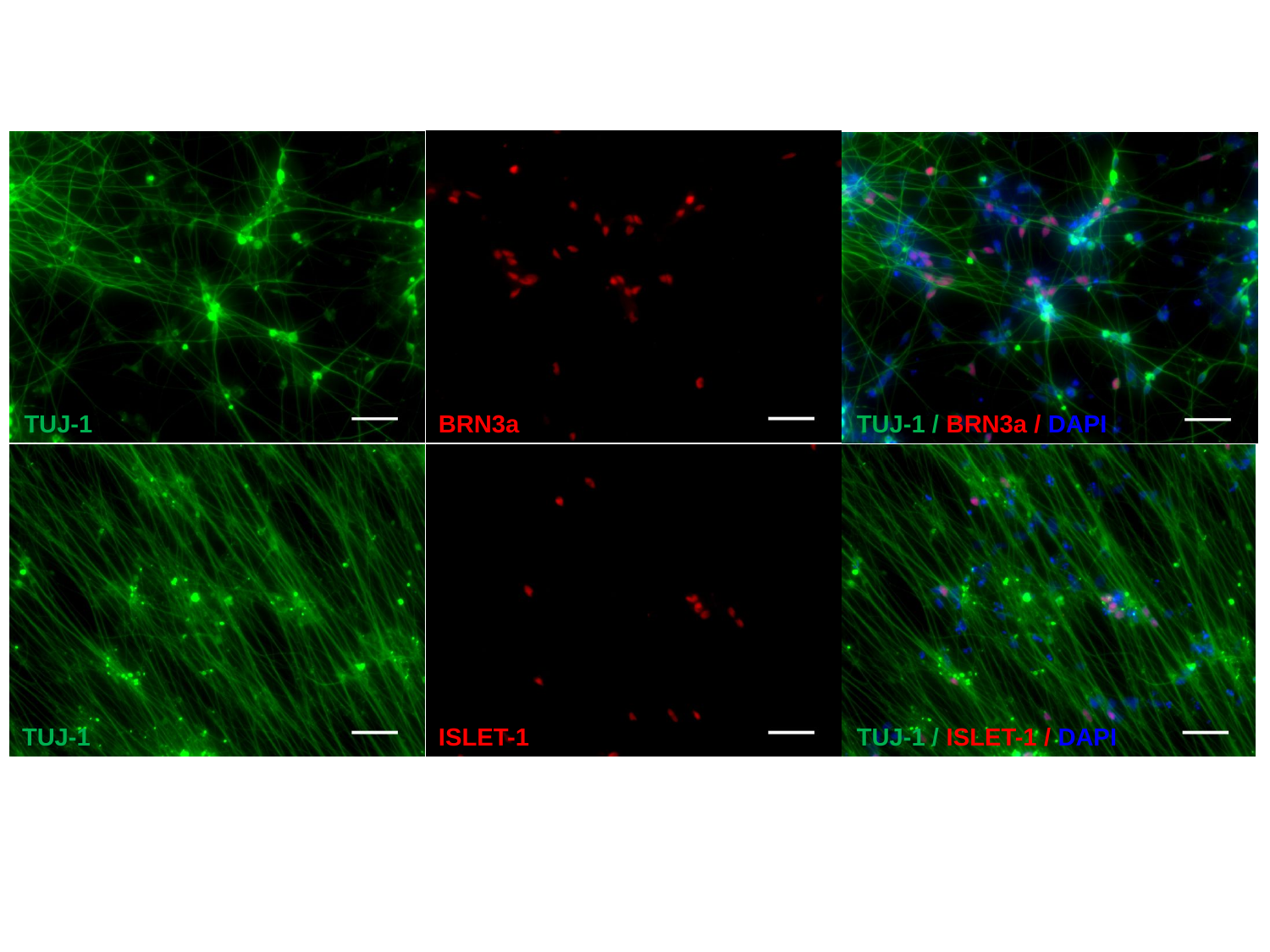

TUJ-1
BRN3a
TUJ-1 / BRN3a / DAPI
TUJ-1
ISLET-1
TUJ-1 / ISLET-1 / DAPI

## Slide 13
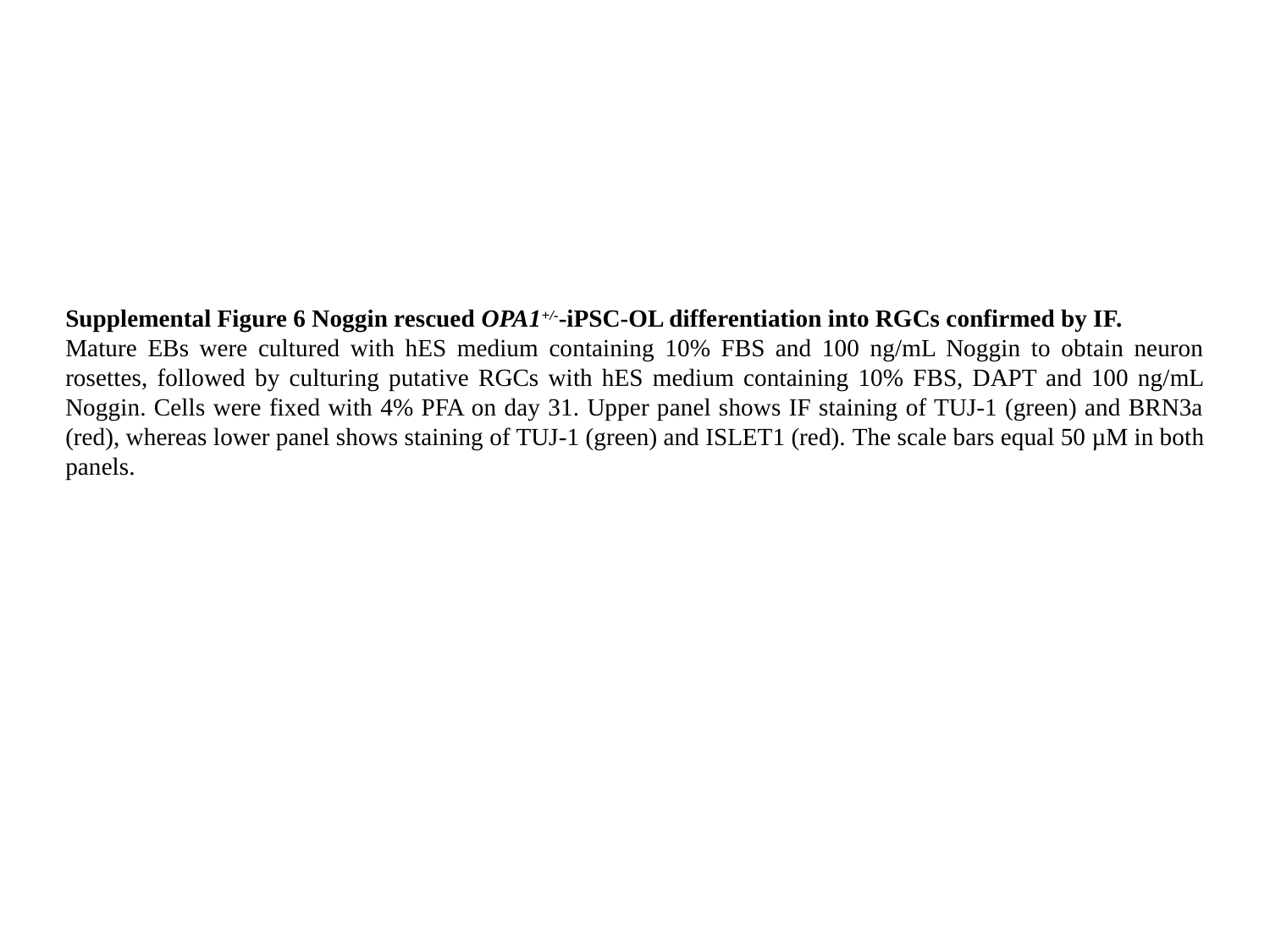

Supplemental Figure 6 Noggin rescued OPA1+/--iPSC-OL differentiation into RGCs confirmed by IF.
Mature EBs were cultured with hES medium containing 10% FBS and 100 ng/mL Noggin to obtain neuron rosettes, followed by culturing putative RGCs with hES medium containing 10% FBS, DAPT and 100 ng/mL Noggin. Cells were fixed with 4% PFA on day 31. Upper panel shows IF staining of TUJ-1 (green) and BRN3a (red), whereas lower panel shows staining of TUJ-1 (green) and ISLET1 (red). The scale bars equal 50 µM in both panels.

## Slide 14
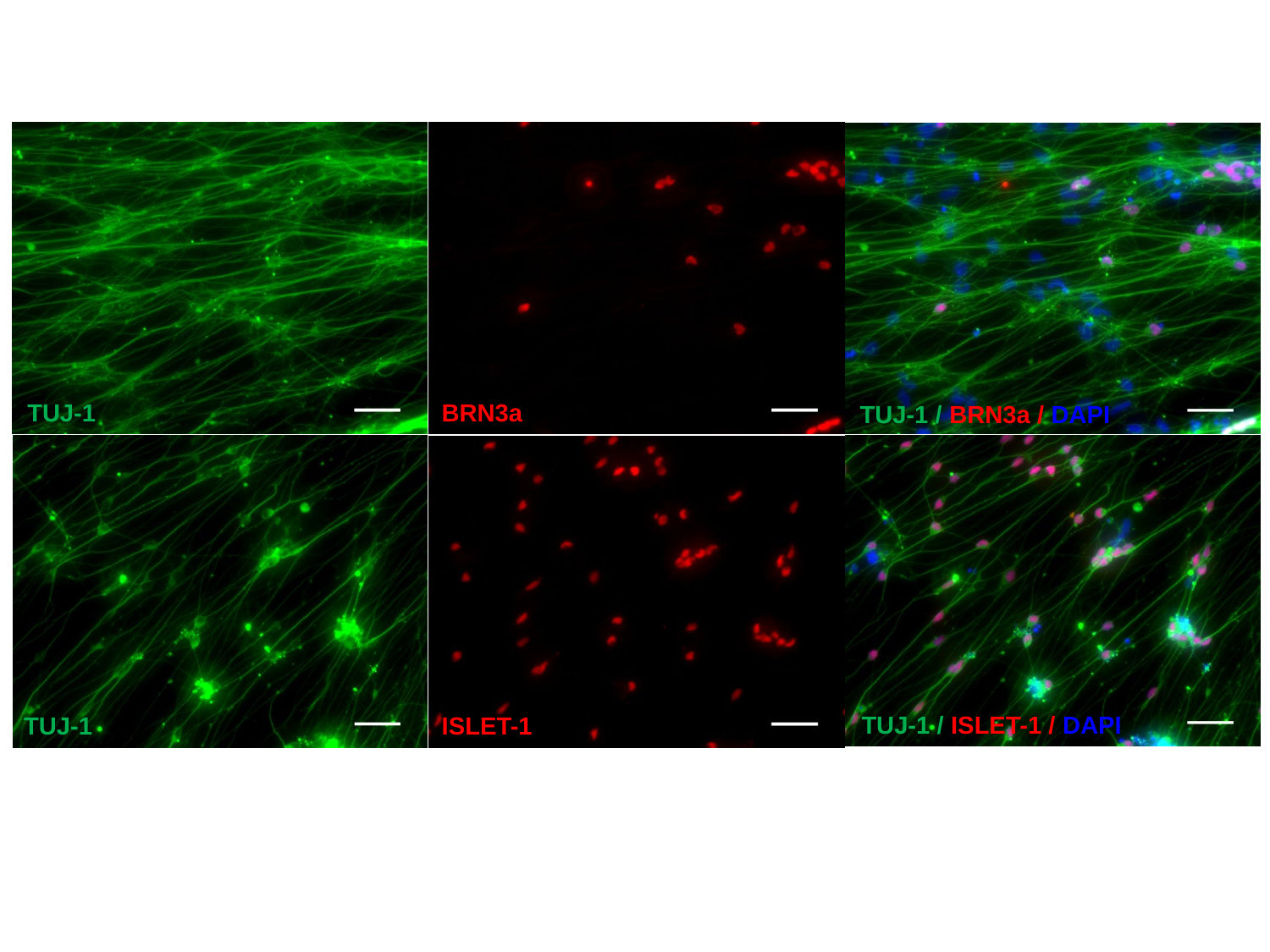

TUJ-1
BRN3a
TUJ-1 / BRN3a / DAPI
TUJ-1 / ISLET-1 / DAPI
TUJ-1
ISLET-1

## Slide 15
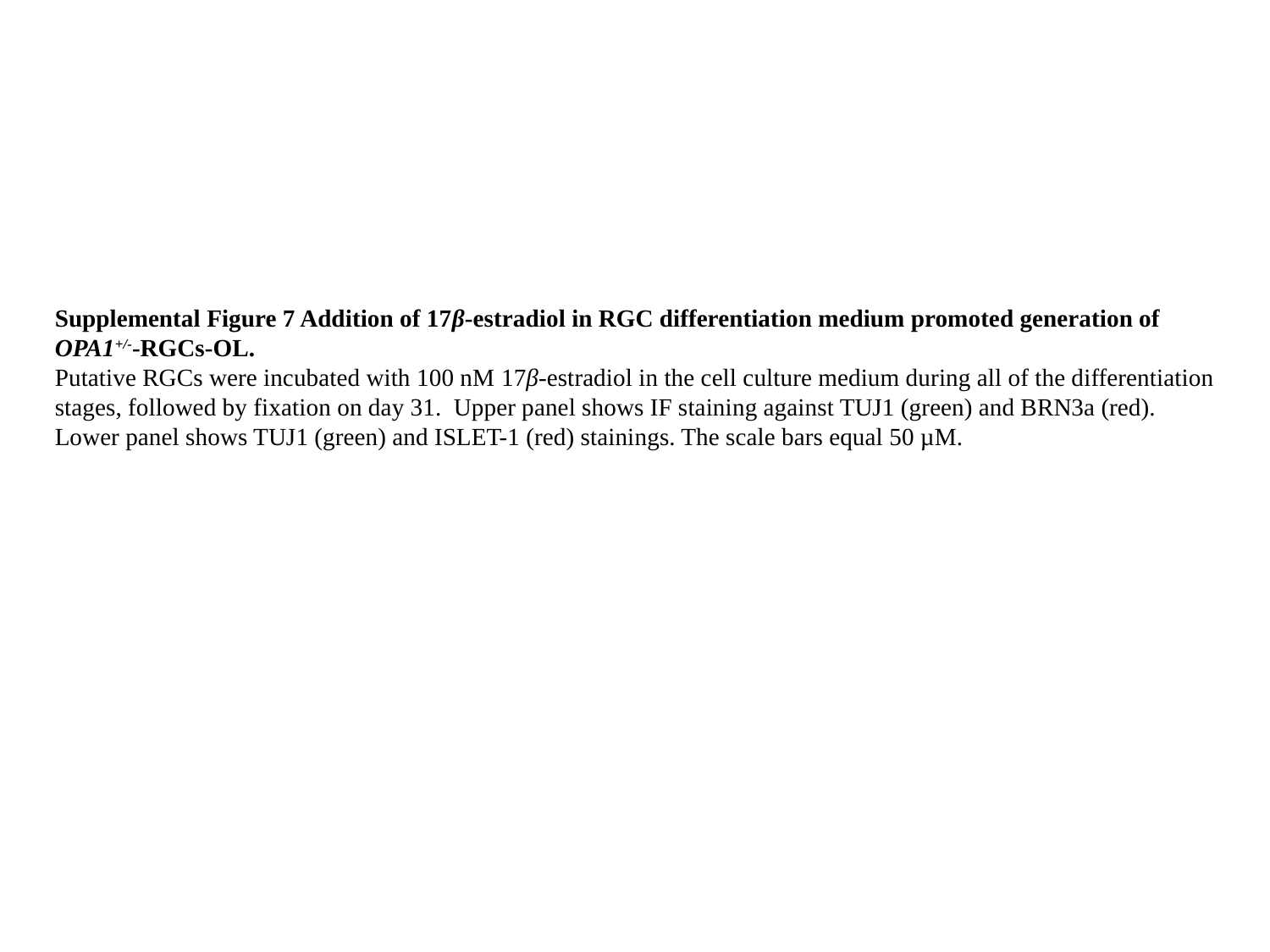

Supplemental Figure 7 Addition of 17β-estradiol in RGC differentiation medium promoted generation of OPA1+/--RGCs-OL.
Putative RGCs were incubated with 100 nM 17β-estradiol in the cell culture medium during all of the differentiation stages, followed by fixation on day 31. Upper panel shows IF staining against TUJ1 (green) and BRN3a (red). Lower panel shows TUJ1 (green) and ISLET-1 (red) stainings. The scale bars equal 50 µM.
